# A Bio-Inspired Adaptive Probability IVYPSO Algorithm with Adaptive Strategy for Backpropagation Neural Network Optimization in Predicting High-Performance Concrete Strength

**DOI:** 10.3390/biomimetics10080515

**Published:** 2025-08-06

**Authors:** Kaifan Zhang, Xiangyu Li, Songsong Zhang, Shuo Zhang

**Affiliations:** 1 School of Computer Science, Hubei University of Technology, Wuhan 430068, China; 13871749360@163.com (K.Z.); 102301187@hbut.edu.cn (S.Z.); 2 Department of Electronic Engineering, Shanghai Jiao Tong University, Shanghai 200240, China; xiangyuli@sjtu.edu.cn; 3 Institute for Environmental Design and Engineering, University College London, London WC1H 0NN, UK

**Keywords:** bio-inspired optimization, high-performance concrete, adaptive probability strategy, ivy algorithm, particle swarm optimization, backpropagation neural network

## Abstract

Accurately predicting the compressive strength of high-performance concrete (HPC) is critical for ensuring structural integrity and promoting sustainable construction practices. However, HPC exhibits highly complex, nonlinear, and multi-factorial interactions among its constituents (such as cement, aggregates, admixtures, and curing conditions), which pose significant challenges to conventional predictive models. Traditional approaches often fail to adequately capture these intricate relationships, resulting in limited prediction accuracy and poor generalization. Moreover, the high dimensionality and noisy nature of HPC mix data increase the risk of model overfitting and convergence to local optima during optimization. To address these challenges, this study proposes a novel bio-inspired hybrid optimization model, AP-IVYPSO-BP, which is specifically designed to handle the nonlinear and complex nature of HPC strength prediction. The model integrates the ivy algorithm (IVYA) with particle swarm optimization (PSO) and incorporates an adaptive probability strategy based on fitness improvement to dynamically balance global exploration and local exploitation. This design effectively mitigates common issues such as premature convergence, slow convergence speed, and weak robustness in traditional metaheuristic algorithms when applied to complex engineering data. The AP-IVYPSO is employed to optimize the weights and biases of a backpropagation neural network (BPNN), thereby enhancing its predictive accuracy and robustness. The model was trained and validated on a dataset comprising 1030 HPC mix samples. Experimental results show that AP-IVYPSO-BP significantly outperforms traditional BPNN, PSO-BP, GA-BP, and IVY-BP models across multiple evaluation metrics. Specifically, it achieved an R^2^ of 0.9542, MAE of 3.0404, and RMSE of 3.7991 on the test set, demonstrating its high accuracy and reliability. These results confirm the potential of the proposed bio-inspired model in the prediction and optimization of concrete strength, offering practical value in civil engineering and materials design.

## 1. Introduction

With the rapid pace of urbanization and ongoing infrastructure development, high-performance concrete (HPC) has found widespread application in high-rise buildings [1], bridge engineering [2], nuclear power plants [3], and other sectors due to its excellent mechanical properties, durability, and workability [4,5]. Among the various performance indicators, concrete compressive strength is a critical factor influencing the safety and durability of engineering structures [6,7,8]. Accurate prediction of the compressive strength of HPC not only ensures structural safety and durability but also optimizes material utilization, reduces waste, and advances sustainable development within the construction sector. Traditionally, compressive strength has been determined through experimental methods. However, this approach is not only time-consuming and costly but also affected by environmental factors and human error, making it challenging to meet the dual demands of both efficiency and accuracy in engineering design [9]. Consequently, the development of an efficient, stable, and highly accurate compressive strength prediction model has become a focal point of research in civil engineering and materials science [10,11,12].

In addressing the challenge of predicting concrete strength, various modeling approaches have been proposed, including multiple linear regression (MLR) [13,14], decision tree (DT) [15,16], support vector machine (SVM) [17,18], and other statistical and machine learning models [19]. However, due to the inherent nonlinearities and multivariate coupling in concrete materials, these methods often struggle to fully capture the complex nonlinear relationships between inputs and outputs, leading to limited prediction accuracy [20]. In contrast, artificial neural networks (ANN) [21], particularly backpropagation neural networks (BPNN) [22,23,24,25], have been widely used in concrete performance prediction because of their strong nonlinear modeling capabilities and adaptability.

Despite the advantages of BPNN in terms of modeling accuracy, their training process relies on the gradient descent optimization method, which is prone to local minima and sensitive to the initialization of weights and network architecture [26]. This sensitivity can adversely affect the model’s predictive accuracy, training efficiency, and its convergence speed [27]. To mitigate these limitations, researchers have explored integrating various intelligent optimization algorithms with BPNN to optimize weights and thresholds, thereby enhancing the model’s global search capability and generalization ability. FZ El-Hassani et al. [28] developed a GA-optimized BPNN model for thyroid disease diagnosis, effectively addressing local minima and slow convergence issues. F Ma et al. [29] applied a GA-optimized BPNN to forecast regional logistics demand, improving prediction accuracy and reducing iteration times. A Abdurrakhman et al. [30] proposed a PSO-optimized adaptive BPNN model to predict and optimize the output power of biogas-fueled generators, achieving high accuracy and effective parameter tuning. Z Wang et al. [31] proposed an XGBoost-assisted OTDBO-BPNN for predicting HPC compressive strength, which achieved superior accuracy by enhancing DBO’s global search and convergence performance through four strategic improvements. In addition, recent research has demonstrated the effectiveness of combining deep learning and 3D point cloud technologies to enhance the performance of intelligent optimization models in human–machine interaction and rehabilitation engineering [32,33], providing further insight for the development of hybrid intelligent frameworks in civil engineering contexts. Meanwhile, recent studies have also emphasized the significance of co-optimizing neural networks using adaptive evolutionary algorithms [34] and the increasing industrial relevance of hybrid nature-inspired population-based optimization methods [35], further supporting the rationale of our proposed hybrid framework.

Inspired by biomimetic principles, where natural systems often balance global exploration with local exploitation to adapt efficiently to complex environments [36,37], this paper introduces a novel BPNN model optimized using an adaptive probability hybrid ivy algorithm (IVYA) [38] and particle swarm optimization (PSO) [39] based on fitness improvement (AP-IVYPSO). The IVYA, drawing inspiration from the natural growth process of ivy plants, mimics biological behaviors to perform efficient local search, while PSO simulates the social behavior of bird flocking for global search. By combining these biomimetic algorithms, the proposed model effectively balances exploration and exploitation, addressing challenges faced by traditional optimization techniques.

The proposed model leverages the global search capabilities of PSO and the local search characteristics of the IVYA. By employing a fitness-based adaptive probability strategy, the model dynamically adjusts the update rules of PSO and IVYA, improving the accuracy, stability, and generalization ability of concrete strength predictions. To evaluate the model’s effectiveness, experiments were conducted using a publicly available concrete dataset and compared with traditional models such as BPNN, PSO-BP, GA-BP, and IVY-BP. The results show that the AP-IVYPSO-BP model outperforms these models across various evaluation metrics, particularly in enhancing the robustness and prediction accuracy of the model. The main contributions of this paper can be summarized as follows:

- We introduce a new hybrid algorithm, AP-IVYPSO, which combines the IVYA and PSO, and incorporates an adaptive probability strategy based on fitness improvement. This biomimetic-inspired approach strikes a balance between global search capability and local search efficiency, effectively addressing the challenges faced by single optimization algorithms—such as getting stuck in local optima, slow convergence, and instability—when dealing with complex nonlinear problems.
- Through comparison experiments with 10 widely recognized optimization (PSO, IVYA, HFPSO, HJSPSO, BOA, WOA, GOOSE, PSOBOA, NSM-BO, and FDB-AGSK.) algorithms on 26 benchmark test functions, AP-IVYPSO demonstrates exceptional optimization capability and high stability.
- When applied to optimize a BP neural network, AP-IVYPSO effectively overcomes the local optima issue typically faced by traditional gradient descent methods, significantly improving the stability of the model. In comparison to existing prediction models, the AP-IVYPSO-BP model outperforms in multiple evaluation metrics (R^2^ = 0.9542, MAE = 3.0404, and RMSE = 3.7991), further validating the superior performance of the proposed approach.

The remainder of this paper is organized as follows: Section 2 introduces the fundamentals of BPNN, PSO, and the IVYA. Section 3 presents the construction and parameter optimization mechanism of the AP-IVYPSO-BP model; Section 4 details the experimental setup, performance evaluation, and comparison with baseline models. Section 5 presents the concluding remarks of this study and delineates prospective directions for subsequent research endeavors.

## 2. Materials and Methods

### 2.1. BP Neural Network

In recent years, the BPNN, as a classical multilayer feedforward neural network, has been widely applied in nonlinear modeling, regression prediction, and classification tasks [40,41]. Its primary strength stems from effectively capturing intricate nonlinear relationships and its strong generalization capability with a relatively interpretable model structure.

BPNN is generally composed of three fundamental parts: a layer for input, one or several hidden layers, and a layer for output, with interconnections defined by adjustable weights. During training, the network processes input data through forward propagation to produce outputs and then applies error backpropagation to iteratively adjust the weights and biases based on the difference between predicted and expected values. This learning mechanism enables the model to progressively minimize prediction errors and extract deep correlations among input features.

Although the use of gradient descent during training may lead to local optima, BPNN remain popular due to their structural flexibility and adaptability. To further improve convergence speed and prediction accuracy, recent studies have integrated BPNNs with intelligent optimization algorithms, resulting in hybrid models with enhanced robustness and global search capabilities.

In this study, the BP neural network is utilized as the foundational model for predicting concrete strength. Instead of relying on the traditional BP backpropagation training method, its weights and biases are optimized through an external global search using a swarm intelligence algorithm.

Each candidate solution $X_{i}$ represents an initial set of network parameters. The swarm intelligence algorithm iteratively adjusts these parameters to minimize the prediction error on the validation dataset. This approach mitigates common issues associated with conventional training techniques, such as local minima and vanishing gradients.

### 2.2. PSO Algorithm

PSO, introduced by Kennedy and Eberhart in 1995, is a population-based metaheuristic inspired by the collective behavior observed in bird flocks and fish schools during foraging. By exchanging information among individuals within a population, the algorithm efficiently balances exploration of the global search space and exploitation of promising local regions to address complex optimization problems [42,43].

In PSO, each individual—referred to as a particle—represents a candidate solution and is characterized by its position vector $X_{i}=[x_{i1},x_{i2},x_{i3},\ldots ,x_{iq},\ldots ,x_{iU}]$ and velocity vector $V_{i}=\left[\begin{array}{c} v_{i1},v_{i2},v_{i3},\ldots ,v_{iq},\ldots ,v_{iU}, \end{array}\right]$, where i= 1, 2, … N, N denotes the population size, q=1,2,…,U, and U denotes the problem dimensionality. At each iteration, a particle’s trajectory is updated based on three fundamental influences:

(1) Inertia term: This retains a particle’s previous velocity, aiding in the continuation of its current search direction and contributing to global exploration.
(2) Cognitive term: This component reflects the particle’s own experience, guiding its movement toward its personal best position $P_{best,i}$.
(3) Social term: Representing collective intelligence, this steers particles toward the globally best-known position $G_{best}$ discovered by the swarm.

In the searching phase, each particle’s location is affected by its personal best position within its vicinity $P_{best,i}$ and the overall best position found by the swarm $G_{best}$ of the entire population.

The formulas for updating the particle’s position and velocity are presented in Equation (1) and Equation (2), respectively.

(1) $$X_{i}^{k+1}=X_{i}^{k}+V_{i}^{k+1},$$

(2) $$V_{i}^{k+1}=\omega V_{i}^{k}+c_{1}\xi_{1}\left(P_{best,i}-X_{i}^{k}\right)+c_{2}\xi_{2}\left(G_{best}-X_{i}^{k}\right),$$

where ω denotes the inertia weight that regulates the trade-off between exploration and exploitation, k denotes the current iteration index, and k=1,2,…,T, T is the maximum number of iteration. The parameters $c_{1}$ and $c_{2}$ are cognitive and social acceleration coefficients, while $\xi_{1}$ and $\xi_{2}$ are uniformly distributed random numbers in the range [0, 1], introducing stochasticity into the search process. The termination condition is defined as reaching the maximum number of iterations, ensuring a balance between convergence quality and computational efficiency.

In the task of predicting concrete compressive strength, PSO is employed to optimize the parameters of the BP neural network. Each particle represents a set of initial parameters for the BP network, with the particle’s trajectory in the search space reflecting the path taken to find the optimal network weights and biases. Each particle’s position vector encodes a set of weights and biases for the BP neural network, and the population consists of several particles that co-evolve within the search space. PSO dynamically adjusts each particle’s velocity and position based on both its individual best position and the global best position, guiding the entire population towards a more optimal solution. The search space is defined by the complex, non-convex space of network parameters, and the fitness function is evaluated using the RMSE on the validation set. Through the iterative particle search process, an optimal set of network parameters can be identified more rapidly, improving prediction accuracy and enhancing the model’s robustness.

**Figure 1:**
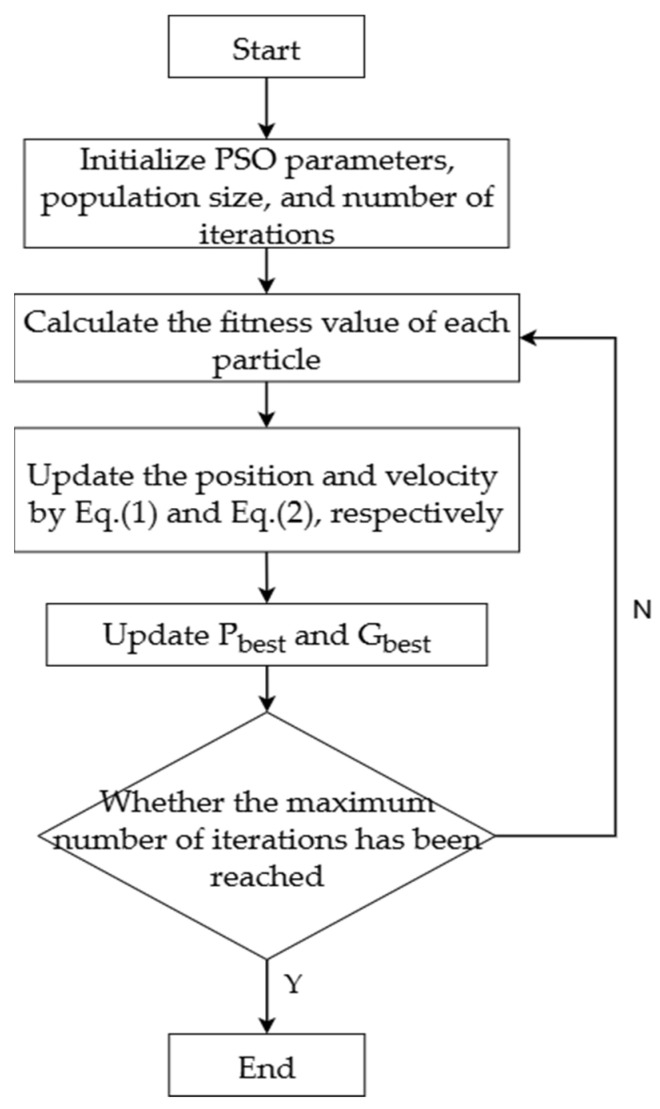
Schematic representation of the PSO algorithm’s workflow.

Figure 1 illustrates the operational workflow of the PSO algorithm.

### 2.3. Ivy Algorithm

The IVYA is an optimization method based on swarm intelligence, drawing inspiration from the adaptive and exploratory nature of vine plants in the natural world. Vines exhibit dynamic behaviors such as climbing, stretching, and expanding as they seek vital resources like sunlight and nutrients. This biological strategy provides a conceptual foundation for tackling global optimization problems. The IVYA emulates several phases of ivy development, including propagation, vertical climbing, and lateral spreading [40]. The algorithm consists of the following four primary stages:

(1) Population Initialization. At the outset, a population of potential solutions is generated. Let N denote the number of individuals and U denote the dimensionality of the optimization problem. Each individual i−th is represented as a U-dimensional vector: $I_{i}=(I_{i1},\ldots ,I_{iU})$, where i∈{1,2,…,N} and N denotes the population size. The entire ivy population is given by $\vec{I}=(I_{1},\ldots ,I_{i},\ldots ,I_{Kpop})$. The initial positions of the individuals are randomly determined within the defined search boundaries using Equation (3):
  (3) $$I_{i}=I_{min}+U(0,1)⊙(I_{max}-I_{min}),i=1,\ldots ,N,$$
  where U(0,1) denotes a U-dimensional vector containing random numbers uniformly distributed between 0 and 1 and ⊙ represents the Hadamard product between two vectors. The search boundaries are defined by $I_{min}$ and $I_{max}$, which represent the lower and upper bounds of the decision space, respectively.

(2) Controlled Growth Dynamics. The population evolves in a structured manner that mimics ivy growth. The rate of growth GV is assumed to vary over time, described via a differential Equation (4):
  (4) $$\frac{dGV(t)}{dt}=\psi \cdot GV(t)\cdot \varphi (GV(t)),$$
  where ψ is a velocity factor, φ is a nonlinear correction term, and GV is the current growth rate. Individual updates are defined using Equation (5):
  (5) $$\Delta {GV}_{i}(t+1)={U(0,1)}^{2}⊙(N(0,1)⊙\Delta {GV}_{i}(t)),$$
  where ${GV}_{i}(t+1)$ and ${GV}_{i}(t)$ are the growth variations at successive time steps and N(0,1) is a Gaussian-distributed random vector.

(3) Sunlight-Driven Adaptation. In nature, vines grow directionally towards light sources, often attaching to structures that support upward movement. This phototropic tendency is captured in the IVYA by encouraging individuals to improve based on their best-performing neighbor. The optimal peer $I_{ii}$ for individual $I_{i}$ is chosen using Equation (6):
  (6) $$I_{ii}=\left\{\begin{array}{c} I_{j-1}^{s},\;\mathrm{if}\;I_{i}=I_{j}^{s}\;\mathrm{and}\;j>1\\ I_{i},\;\mathrm{if}\;I_{i}=I_{best} \end{array},\right.$$
  where the variable j represents the index of individual $I_{i}$ in the population sorted by fitness from best to worst, with j∈{1,2,…,N}. The selection procedure is as follows:

- Sort the population according to fitness values in descending order, producing a sorted sequence $I_{1}^{s},I_{2}^{s},\ldots ,I_{N}^{s}$.
- Find the position j of individual $I_{i}$ in this sorted list.
- If j>1, the optimal peer $I_{ii}$ is the immediate better-ranked neighbor $I_{j-1}^{s}$.
- If j=1, meaning $I_{i}$ is the current best individual $I_{best}$, then $I_{ii}=I_{best}$ itself.

This selection ensures that each individual learns from the nearest superior peer in the fitness ranking, mimicking the natural tendency of vines to grow towards more favorable structures.

The new state of individual $I_{i}$ is calculated with Equations (7) and (8):

(7) $$I_{i}^{new1}=\left|K(1,U)\right|⊙(I_{ii}-I_{i})+I_{i}+K(1,U)⊙\Delta {GV}_{i},i=1,2,3,\ldots ,K,$$

(8) $$\Delta {GV}_{i}=\left\{\begin{array}{c} I_{i}⊗(I_{max}-I_{min}),\mathrm{Iter}=1\\ {U(0,1)}^{2}⊙(N(0,1)⊙\Delta Gv_{i}),\mathrm{Iter}>1 \end{array},\right.$$

where $\left|K(1,U)\right|$ represents a vector with absolute values of the normal distribution components, enhancing diversity and exploration in the search space.

**Figure 2:**
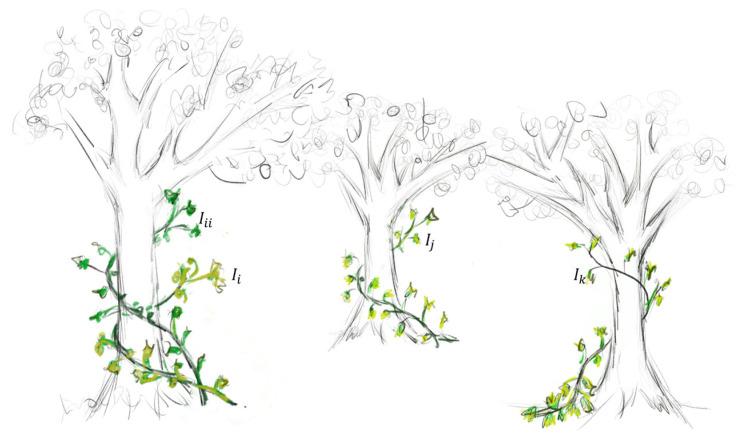
The individual $I_{i}$ selects the nearest and most influential neighbor $I_{ii}$ within the population.

To replicate this behavior, the algorithm allows each individual $I_{i}$ to identify and refer to the nearest neighbor $I_{ii}$ with superior fitness as a guide for its own evolution process. This mechanism, which mimics the natural tendency of vines to grow toward favorable conditions, is illustrated in Figure 2.

The last primary stage is as follows:

(4) Growth behavior and evolutionary adjustment. Once an individual $I_{i}$ has explored the global space and located its closest high-quality neighbor $I_{ii}$ it proceeds to align its search direction toward the current global best solution $I_{best}$. This phase emphasizes exploiting the local region around $I_{best}$ to refine the solution, as formulated in Equations (9) and (10).

(9) $$I_{i}^{new}=I_{best}⊙(U(0,1)+N(0,1)⊙\Delta {GV}_{i}),$$

(10) $$\Delta {GV}_{i}^{new}=I_{i}^{new}⊗(I_{max}-I_{min}),$$

In this study, the IVYA is employed to optimize the initial weights and biases of the BP neural network, thereby enhancing the accuracy of the network’s prediction of concrete compressive strength. In practice, each IVYA individual is represented as a vector, which encodes a complete set of neural network weights and bias parameters. The population consists of multiple such individuals, and the search space is defined by the high-dimensional, non-convex error function space associated with the BP network parameters. IVYA efficiently explores this complex space through mechanisms such as simulating vine extension, selecting growth nodes, and perturbing local solutions. In each iteration of the algorithm, a new growth node is generated, corresponding to a new configuration of neural network parameters, which is then used to construct the corresponding BP model and assess its prediction performance on both the training and validation datasets. The fitness function is defined as the root mean square error (RMSE) on the validation set, with the goal of minimizing this error to improve the network’s generalization ability. This approach effectively mitigates common challenges in neural network training, such as vanishing gradients and local optima.

**Figure 3:**
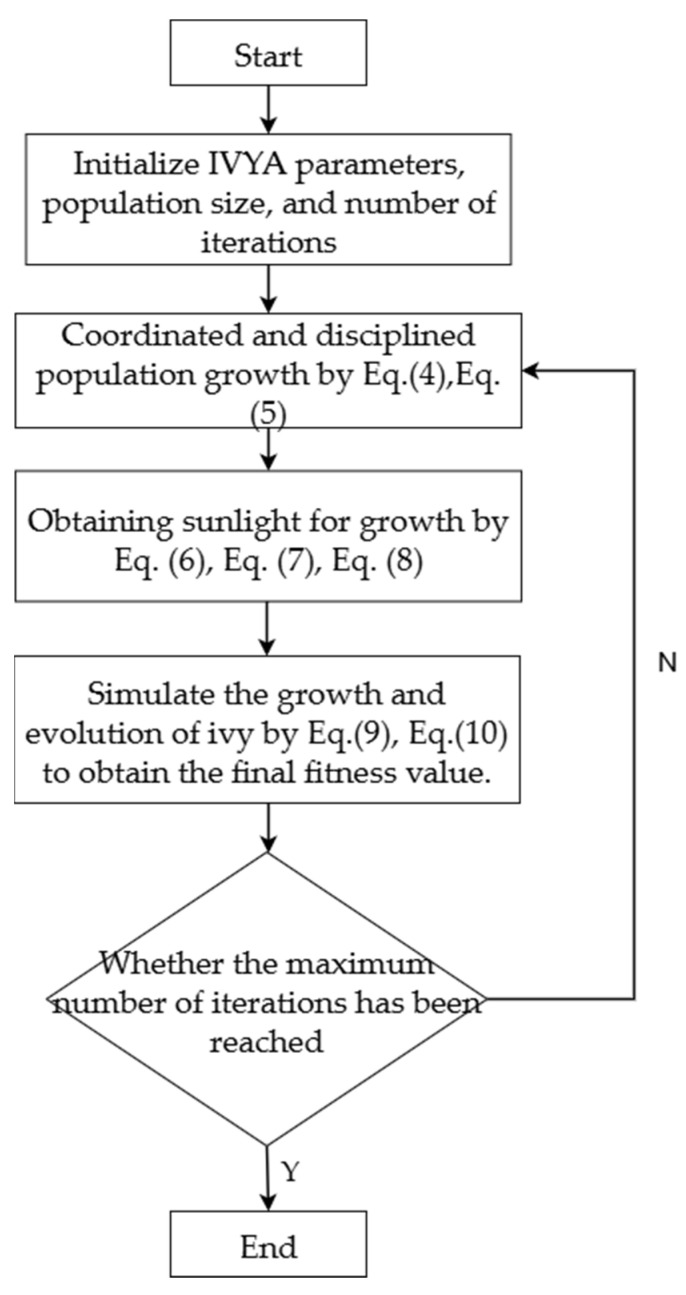
Schematic representation of the ivy algorithm’s workflow.

The search process is terminated when the maximum number of iterations is reached. Figure 3 illustrates the procedural flow of the ivy algorithm.

## 3. Construction and Parameter Optimization Mechanism of the AP-IVYPSO-BP Model

This section presents a detailed description of the construction and parameter optimization mechanism of the AP-IVYPSO-BP model. To address the challenges faced by traditional BPNN, including issues of local optima and low convergence efficiency during training, this paper introduces an AP-IVYPSO to optimize the BPNN. The proposed model integrates the global search capabilities of PSO with the local search features of the IVYA. Unlike simple hybrid strategies that alternate update steps or linearly weight two algorithms, the AP-IVYPSO model adaptively switches between PSO and IVYA based on a fitness-driven probability function. This mechanism ensures seamless cooperation between the fast exploration of PSO and the refined exploitation of IVYA. Through an adaptive probability mechanism based on fitness improvement, the model dynamically adjusts the update strategies of PSO and IVYA. The strategy mimics the natural adaptation behavior observed in vine plants exposed to sunlight, which selectively grows towards better environmental conditions—an inspiration that guides this adaptive optimization framework. This combination effectively enhances the accuracy, stability, and generalization ability of concrete compressive strength prediction.

Specifically, in addressing the typical regression problem of predicting concrete strength, the BP network’s training process is redefined as a parameter optimization problem, where the AP-IVYPSO algorithm directly updates the parameters within the network architecture through iterative adjustments.

### 3.1. Implementation Mechanism of AP-IVYPSO

To improve the dynamic balance between global search capability and local refinement ability in complex engineering problems, this paper introduces an intelligent optimization algorithm based on an adaptive probability-guided mechanism, named AP-IVYPSO. The method incorporates an adaptive probability control mechanism into the core iteration process, dynamically adjusting the tendency of individuals to select search strategies at various stages. This facilitates the complementary coordination between the global exploration ability of PSO and the local fine search capability of the IVYA. By guiding the switching of search strategies at different stages of the evolutionary process, AP-IVYPSO effectively enhances both the global convergence performance and the local convergence accuracy of the algorithm. As a result, it achieves adaptive adjustment of the search direction and a seamless integration of multi-stage search behaviors. The method demonstrates excellent adaptability and robustness, making it particularly suitable for solving complex, nonlinear, and multimodal engineering optimization problems.

#### 3.1.1. Adaptive Probability-Guided Mechanism

In the AP-IVYPSO algorithm, the core idea is to dynamically determine whether the current individual will use the PSO update strategy or the IVYA update strategy in each iteration, based on an adaptive probability control mechanism. This mechanism is calculated using the Equation (11):

(11) $$P_{adapt}=exp\left(-\frac{5t}{T}\right),$$

where t is the current iteration number and T is the maximum number of iterations. $exp\left(x\right)$ represents the natural exponential function, which is the exponential function with the base of the natural constant e≈2.71828. The term $\frac{5t}{T}$ represents the position of the current process in the iteration. Multiplying by 5 is used to control the rate of decay, which likely refers to how quickly the influence of certain parameters reduces over time in the algorithm.

Each individual generates a random number U(0,1) during the iteration and selects the search strategy based on the following rule:

- If $U(0,1)<P_{adapt}$, the PSO update strategy is selected;
- Otherwise, the IVYA update strategy is chosen.

The function of this mechanism is as follows:

- Early iterations (t ≪ T): at this stage, ${\;P}_{adapt}\approx 1$, indicating that individuals are more likely to adopt the PSO strategy, which enhances global search capabilities by exploring a wider solution space.
- Later iterations (t ≈ T): at this stage, ${\;P}_{adapt}\approx 0$, at which point the algorithm shifts to using the IVYA strategy, emphasizing local refinement and fine-tuning of solutions.

The core innovation of this mechanism is that it quantifies the trade-off between global and local search through a decaying probability function. Early in the optimization, a high $P_{adapt}$ favors PSO, enabling the swarm to explore broadly and avoid premature convergence. As the search progresses, $P_{adapt}$ decreases, making IVYA more likely to dominate, which improves fine-tuning and convergence precision.

This approach is grounded in adaptive optimization theory, where dynamically adjusting exploration and exploitation according to convergence state is a proven strategy for avoiding local optima in multimodal problems.

#### 3.1.2. Global Search Strategy with PSO

When the condition $U(0,1)<P_{adapt}$ is met, individual i will adopt the standard PSO strategy to update its position and velocity, as specified in Equations (1) and (2). This strategy is guided by the individual’s best historical experience and the global best information from the entire population, offering strong global search capabilities and parallel information sharing.

During the early iterations, with a high value of $P_{adapt}$, individuals are more likely to adopt the PSO strategy. This encourages the population to quickly expand the search space, avoid local optima, and enhance both the global exploration ability and search diversity of the algorithm. At this stage, the algorithm can gather richer search information on a global scale, which provides a solid foundation for the later local search phase. This leads to improved overall optimization efficiency, allowing the model to refine solutions more effectively in subsequent stages.

#### 3.1.3. Local or Global Search Strategy with IVYA

When $U(0,1)>P_{adapt}$, the individual adopts the IVYA strategy, with its search behavior guided by the “vine disturbance mechanism” to achieve either local development or global exploration.

First, an adaptive disturbance threshold is generated as Equation (12):

(12) $$\beta_{1}=1+{U(0,1)}^{2},$$

where U(0,1) refers to a random number uniformly distributed in the interval [0, 1]. If the current individual’s fitness $f_{i}$ is less than $\beta_{1}G_{best}$, the individual is considered to be in a potentially optimal region, and a local search is performed as Equation (13):

(13) $$X_{i}^{new}=X_{i}+|N(0,1)|⊙(X_{j}-X_{i})+N(0,1)⊙{GV}_{i},$$

where $X_{j}\;$ refers to another individual randomly selected from the population and $N\left(0,1\right)$ represents a standard normal random variable.

Otherwise, global search is performed as Equation (14):

(14) $$X_{i}^{new}=G_{best}⊙(U(0,1)+N(0,1)⊙{GV}_{i}),$$

This strategy effectively balances exploration and exploitation. Under the control of the adaptive probability mechanism, the algorithm adjusts the search behavior at different stages of the optimization process, using more local search behavior in promising areas and broader global search behavior when exploring new regions. This adaptive mechanism ensures that the algorithm can efficiently explore the solution space while avoiding local optima.

The IVYA’s role here is crucial: its vine disturbance mechanism simulates a localized perturbation process where solutions ‘grow’ along promising paths but with stochastic fine-scale adjustments. This is particularly important to compensate for PSO’s tendency to converge prematurely in high-dimensional, separable search spaces.

Moreover, the IVYA search contributes both local and global search modes. If the solution is near a potential optimum, the local mode is triggered to exploit it further. If the solution is suboptimal, global search provides a chance to escape poor regions. This behavior, governed by the disturbance threshold β_1_, ensures search robustness across various fitness landscapes.

#### 3.1.4. GV Update and Greedy Selection Mechanism

The vine disturbance variable GV controls the search disturbance amplitude in the IVYA strategy, and its update mechanism is given by Equation (15):

(15) $$GV=GV\cdot ({U(0,1)}^{2}\cdot N(0,1))$$

This update rule mimics the natural mutation and contraction behavior observed in vine growth, enabling the search process to be dynamically adjusted. By introducing controlled randomness, this mechanism enhances the algorithm’s capability to escape local optima. The update procedure involves checking and correcting the new position to ensure it stays within predefined boundaries, evaluating its fitness based on the objective function, and then applying a greedy selection mechanism. If the new position’s fitness is better than the current one, it replaces the old solution. Furthermore, if it outperforms the individual’s historical best or the global best solution, the respective records are updated accordingly. This approach guarantees effective evolution of the population each generation while maintaining diversity, which is essential for preventing premature convergence and improving overall search performance.

#### 3.1.5. Time Complexity Analysis

The overall time complexity of the AP-IVYPSO algorithm proposed in this paper can be estimated based on its iterative structure. Let N represent the population size, U the problem dimension, and T the maximum number of iterations. In each iteration, every individual performs either a PSO or IVY update operation, determined by an adaptive probability strategy aimed at improving fitness. Both update operations involve vector calculations of dimension U and a single evaluation of the objective function.

The primary computational costs per generation include updating the position and velocity of each individual, perturbing the vine variable GV, and calculating the fitness for all individuals. Assuming that the complexity of evaluating the objective function is O(U), the computational complexity for each iteration is O(N⋅U). Therefore, the total time complexity for the entire algorithm is given by Equation (16):

(16) O(T⋅N⋅U)

This complexity increases linearly with the number of iterations, population size, and problem dimension, which ensures good scalability for practical engineering optimization problems. Notably, this paper balances algorithm performance with computational efficiency. In the experiments, the population size N is consistently set to 30, which helps ensure the algorithm achieves high computational efficiency while maintaining its optimization capabilities.

### 3.2. Performance Testing of AP-IVYPSO

To validate the performance and effectiveness of AP-IVYPSO, this study selected 26 widely used benchmark test functions [44,45,46,47], which include 15 single-peak test functions (f1–f15) and 11 multi-peak test functions (f16–f26). Single-peak test functions have a single global optimum and relatively simple search spaces. The detailed information about the test functions can be found in Table 1. The optimization process mainly focuses on evaluating the algorithm’s convergence speed and accuracy. With no local optima to interfere with the search, single-peak functions are ideal for testing the algorithm’s local search capability and convergence stability.

In contrast, multi-peak test functions feature multiple local optima and one or more global optima, creating a more complex search space structure. These functions test the algorithm’s ability to escape from local optima and assess its global search performance, making them useful for evaluating the algorithm’s robustness and exploration capabilities in complex environments.

By testing both single-peak and multi-peak functions, we can comprehensively measure the algorithm’s performance across different problem types. Additionally, the 30 independent runs of AP-IVYPSO were compared with the results of eight other widely recognized, high-performance optimization algorithms: PSO, IVYA, HFPSO [48], HJSPSO [49], BOA [50], WOA [51], GOOSE [52], PSOBOA [53], NSM_BO [54], and FDB_AGSK [55]. The parameter configurations for each algorithm can be found in Table 2. Three numerical evaluation metrics were used: the best fitness value, the average value, and the standard deviation, with the formulas described as Equation (17) through (19):

(17) $$Best=\underset{1\leq i\leq R}{min}\;f_{i},$$

(18) $$Avg=\frac{\sum_{i=1}^{R}\;(f_{i})}{R},$$

(19) $$Std=\sqrt{\frac{1}{R-1}\sum_{i=1}^{R}\;(f_{i}-Avg{)}^{2}},$$

where R represents the number of runs, set to 30 in this case. The maximum number of iterations for the algorithms is set to 500.

All experiments were performed on a Windows 10 operating system, equipped with a 32 GB of RAM and Intel (R) Core (TM) i9-14900KF processor (3.10 GHz), using the Matlab R2023a environment.

#### 3.2.1. Numerical Results Analysis

Table 3 presents the best fitness values and rankings achieved by AP-IVYPSO and the other eight algorithms across 26 test functions. AP-IVYPSO achieved the best fitness value on 21 (f1–f4, f6, f8–f20, f24–f26) test functions, demonstrating its strong accuracy and local search capability. However, on functions f22 and f23, it ranked 10 and 4, respectively, indicating average performance.

Table 4 shows the average fitness values, standard deviations, and rankings of AP-IVYPSO and the other eight algorithms across 26 test functions. AP-IVYPSO achieved the best average fitness value on 23 (f1–f6, f8–f21, f24–f26) test functions, highlighting its excellent global search capability and stability. On f7, f22, and f23, however, it ranked 8, 8, and 6, respectively, reflecting average performance compared to the best-performing algorithms on these functions.

#### 3.2.2. Convergence Curve Analysis

Appendix A.1 presents the convergence curves of the proposed AP-IVYPSO-BP algorithm and comparison algorithms, showing how the average fitness values of the benchmark test functions change with the number of objective function evaluations. This provides a more comprehensive representation of the optimization process and overall performance of each algorithm. The x-axis indicates the number of objective function evaluations (with a maximum of 15,000), while the y-axis displays the average fitness values obtained from 30 independent runs, thereby minimizing the random fluctuations that could arise from a single trial.

The figure reveals that the AP-IVYPSO algorithm exhibits faster convergence and superior average performance across 18 test functions (f1–f4, f8–f9, f11, f13–f20, and f24–f26), highlighting its robust global optimization capability. However, on functions f5, f7, f22, and f23, certain comparison algorithms achieved better optimization results, suggesting that these algorithms also demonstrate strong adaptability to specific problem instances.

#### 3.2.3. Friedman Ranking and Wilcoxon Signed-Rank Test

To comprehensively evaluate the performance of the proposed AP-IVYPSO algorithm on 26 test functions, the Friedman test was employed to rank the nine algorithms. Based on the Friedman test scores, AP-IVYPSO achieved the lowest average rank (1.8587), securing first place and demonstrating the best overall performance. IVY and FDB-AGSK followed in second and third place, respectively. The traditional PSO was ranked 10, while GOOSE ranked the lowest, in 11 place. These ranking results further validate the significant advantage of the AP-IVYPSO algorithm in terms of optimization quality and stability. The Friedman ranking of each algorithm is shown in Table 5.

To further validate the significant advantage of the proposed AP-IVYPSO algorithm on multiple benchmark test functions, the Wilcoxon signed-rank test was performed for paired comparisons between AP-IVYPSO and the other eight comparison algorithms. The significance level for the test was set to α = 0.05. The results showed that the *p*-values between AP-IVYPSO and all other algorithms were smaller than the significance level of 0.05. This indicates that, in statistical terms, there are significant differences between AP-IVYPSO and all the comparison algorithms, further confirming the superior optimization performance of the proposed algorithm. The Wilcoxon signed-rank test results for AP-IVYPSO and the other eight algorithms are displayed in Table 6.

In conclusion, AP-IVYPSO has shown outstanding performance across all comprehensive tests, proving itself to be a powerful algorithm.

### 3.3. BPNN Model Parameter Optimization

**Figure 4:**
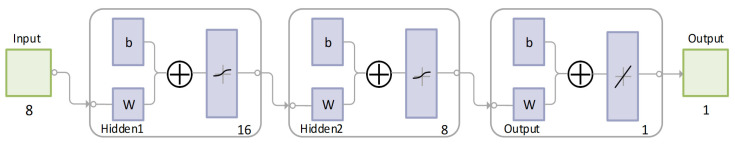
Basic structure diagram of AP-IVYPSO-BP model.

The AP-IVYPSO-BP model utilizes a feedforward neural network for predicting concrete compressive strength. The network consists of an input layer, two hidden layers, and an output layer. The basic structural diagram of the AP-IVYPSO-BP model is shown in Figure 4. The input layer includes features related to the concrete mix, while the output layer predicts the compressive strength of the concrete. The network’s weights and biases are optimized using the AP-IVYPSO algorithm to enhance the accuracy of the predictions.

The network is trained by minimizing the mean squared error (MSE) between the predicted values and the actual values. The AP-IVYPSO algorithm is used iteratively to optimize the weights and biases of the network, with each particle’s position representing a specific set of neural network parameters.

The algorithm begins by initializing the positions and velocities of the particle swarm and evaluating each particle’s fitness based on the performance of the neural network on the training set. The AP-IVYPSO algorithm then optimizes the model through an adaptive probability mechanism based on fitness improvement. In each optimization iteration, the particle positions are updated according to the probabilities of the PSO or IVYA strategies. The optimization process continues until the maximum number of iterations is reached or a convergence criterion is satisfied. Once the optimization is complete, the optimal parameters identified are used to initialize the BPNN. The network is then trained on the training set using the backpropagation algorithm, which aims to minimize the prediction error.

The reason for choosing AP-IVYPSO to optimize the BPNN lies in the complementary nature of the two algorithms: PSO enables efficient global exploration of the neural network’s high-dimensional weight space, while IVYA introduces precise local search adjustments through its disturbance mechanism. This helps fine-tune the network parameters for better predictive accuracy.

Moreover, the AP-IVYPSO’s adaptive switch strategy ensures that early optimization focuses on avoiding poor minima, while later optimization emphasizes convergence around strong solutions. This is highly suitable for training BPNNs, which are known to suffer from poor initialization and gradient-based convergence issues.

### 3.4. Summary

The AP-IVYPSO-BP model integrates the global search ability of PSO and the local optimization capabilities of IVYA. By utilizing the adaptive probability mechanism, the model dynamically selects the most suitable optimization strategy, effectively balancing global and local search efforts. This balance significantly enhances the accuracy and stability of concrete compressive strength predictions. The next section will discuss the experimental setup, performance evaluation, and comparisons with benchmark models.

## 4. Experimental Evaluation and Result Interpretation

### 4.1. Dataset Overview for Experimentation

This study employs a dataset derived from the high-performance concrete compressive strength experimental dataset in the UCI Machine Learning Repository [56]. This dataset is widely utilized in research on the prediction of HPC properties and holds significant representativeness and practical value [57]. The dataset comprises 1030 samples, each with eight input variables and one output variable. The input variables include the following: cement, fly ash, blast furnace slag, water, superplasticizer, age, fine aggregate, and coarse aggregate, while concrete compressive strength serves as the output variable. All input variables, with the exception of Age, are quantified in kilograms per cubic meter (kg/m^3^); age is measured in days, while compressive strength is expressed in megapascals (MPa).

The data were collected from controlled laboratory experiments simulating realistic HPC mix designs, ensuring high data reliability and consistency. The samples cover a broad range of mixture proportions and curing ages, which effectively represent the variability encountered in practical engineering scenarios. This diversity in the dataset allows for robust modeling of the nonlinear and complex relationships between mixture components and compressive strength.

In this study, each sample represents a unique high-performance concrete mix design, and the nonlinear mapping between the eight-dimensional input variables and compressive strength output defines a high-dimensional, non-convex search space. This space lacks gradient information and contains numerous local optima, often metaphorically referred to as an “inhospitable environment.” Both IVYA and PSO are applied to this space to search for the optimal parameters of the neural network.

Specifically, each individual in the population represents a set of initial weights and biases for the BP neural network, which are encoded as real-valued vectors. The optimization algorithm’s goal is to minimize the model’s prediction error on the training or validation set. By initializing the BP neural network with the weights corresponding to each individual, the network is trained on the normalized high-performance concrete dataset, and its performance on the test set is evaluated to assess the fitness of each individual. In this manner, the dynamic search process of swarm intelligence is directly applied to the optimization of the concrete strength prediction parameters.

**Figure 5:**
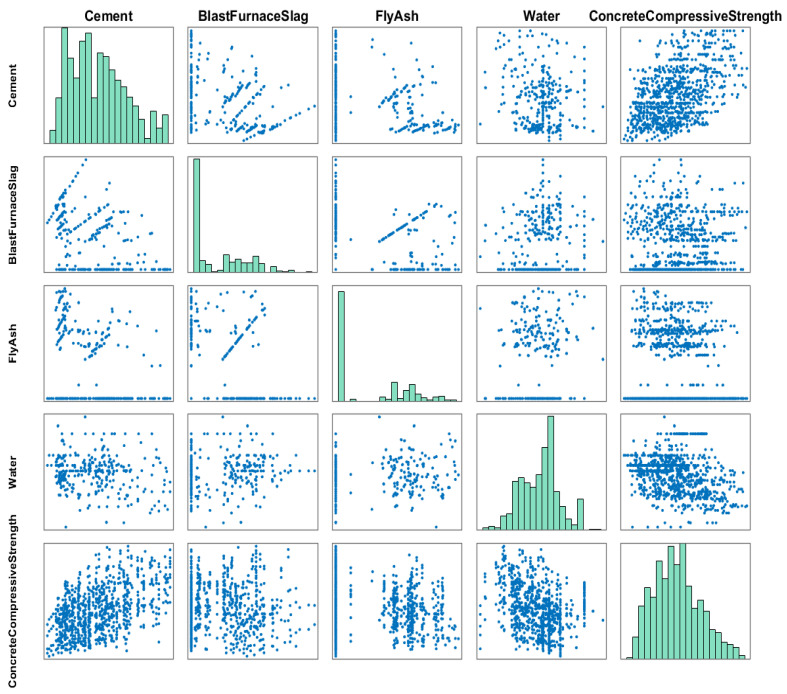
The relationship graph between concrete strength and the initial 4 input features: cement, water, fly ash, and blast furnace slag.

**Figure 6:**
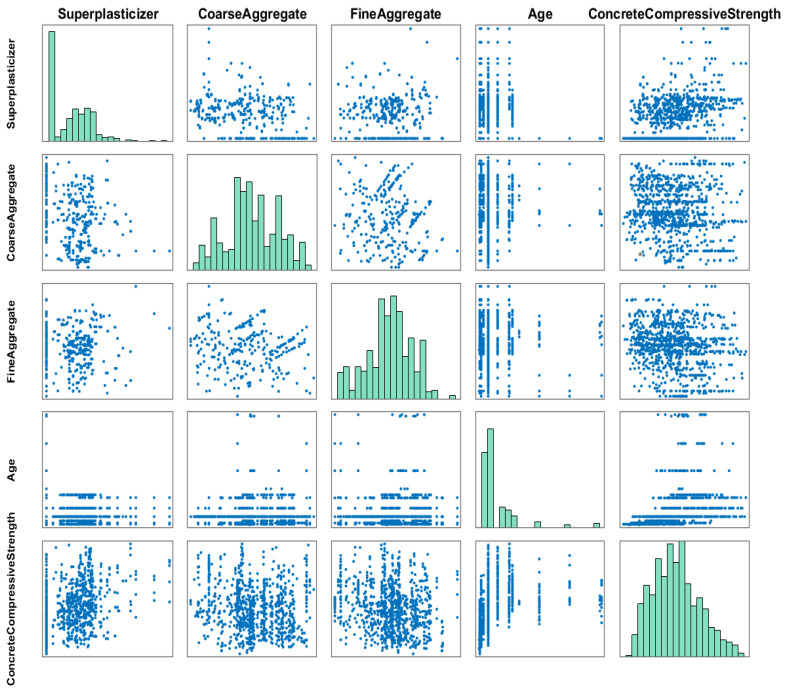
The relationship graph between concrete strength and the final 4 input features: high-efficiency water reducer, age, fine aggregate, and coarse aggregate.

The compressive strength of HPC exhibits a distinct nonlinear relationship with the composition of the mixture, and this complexity is visually illustrated in Figure 5 and Figure 6. Detailed information about the input features is presented in Table 7, facilitating a thorough understanding of their characteristics. By using this data for neural network training and swarm intelligence-based optimization, the algorithmic strategy is not generic or abstract but is specifically customized to model the highly nonlinear strength behavior of concrete.

**Figure 7:**
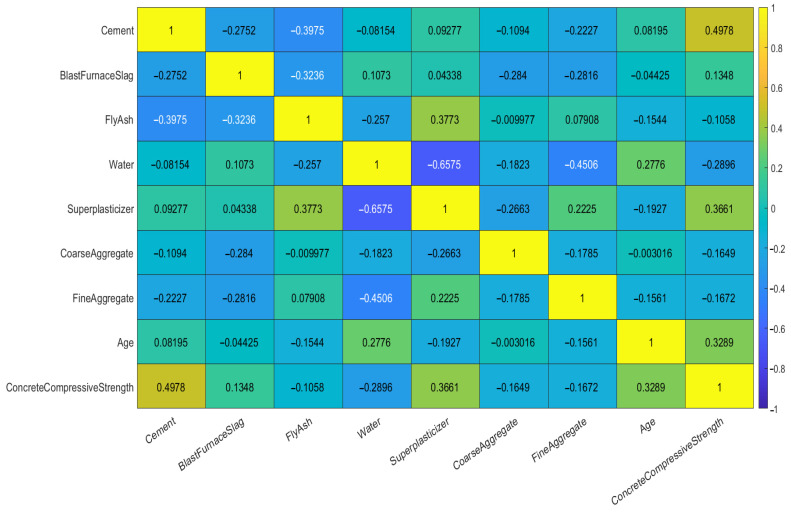
The Pearson correlation matrix for each variable.

To better reveal the interactions between variables, this paper employs the Pearson Correlation Coefficient to analyze the correlations among the input variables and between input variables and output variable. The coefficient ranges from −1 to 1, where values near 0 suggest a weak correlation, values approaching 1 indicate a strong positive correlation, and values close to -l represent a strong negative correlation. The resulting Pearson correlation matrix is displayed in Figure 7, from which the correlation patterns between variables can be discerned. The results demonstrate that the influence of various factors on strength follows distinct and significant correlation patterns.

**Figure 8:**
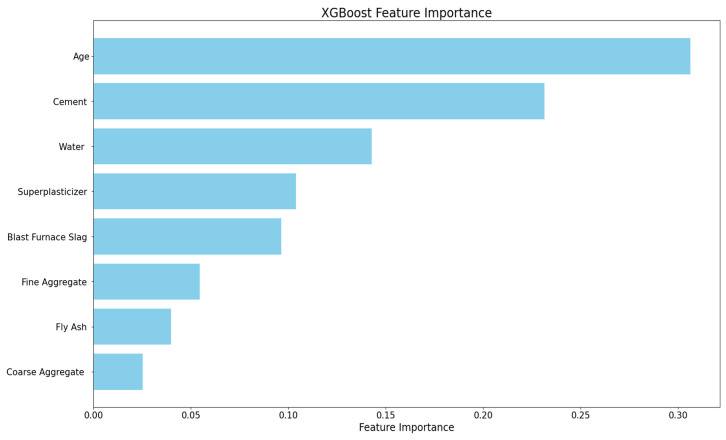
Ranking of feature importance.

Furthermore, to quantitatively evaluate the influence of each input variable on the compressive strength of HPC, the XGBoost algorithm was employed within a Python 3.8 computational environment. The resulting feature importance rankings, as illustrated in Figure 8, reveal that curing age is the most critical predictor, with cement and water con-tent following closely behind. In contrast, fly ash and coarse aggregate have relatively smaller impacts.

To ensure stable convergence during the model training process and eliminate disparities in the magnitudes of different feature variables, the input features were normalized to the [0, 1] range using min-max normalization, while the output targets were standardized using the Z-score method. This preprocessing step enhances the training efficiency and prediction accuracy of the neural network, while mitigating potential convergence issues or instability arising from significant differences in data scales.

In this study, the primary research goal is to accurately predict the compressive strength of high-performance concrete based on its mix design parameters. To achieve this, a hybrid prediction model named AP-IVYPSO-BP is proposed, in which the AP-IVYPSO is employed to optimize the initial weights and biases of a BPNN. By combining global search capability with nonlinear learning, this model aims to address the complex mapping relationship between concrete components and compressive strength, thereby improving prediction precision.

### 4.2. Performance Evaluation Metrics

To assess the predictive performance of the proposed algorithmic model, this study utilizes three widely used evaluation metrics: the coefficient of determination ($R^{2}$), mean absolute error (MAE), and root mean squared error (RMSE). RMSE and MAE assess the differences between actual values and predicted value; whereas lower values signify better prediction accuracy, $R^{2}$ quantifies the degree of correlation between the model’s predicted results and the observed outcomes. Its value ranges from 0 to 1, with values approaching 1 indicating superior model performance and stronger predictive accuracy. The detailed computational expressions are outlined in Equation (20) through (22):

(20) $$R^{2}=1-\frac{\sum_{i=1}^{N}\;(y_{i}-{\hat{y}}_{i}{)}^{2}}{\sum_{i=1}^{N}\;(y_{i}-\bar{y}{)}^{2}}\;f_{i},$$

(21) $$MAE=\frac{1}{N}\sum_{i=1}^{N}\;|{\hat{y}}_{i}-y_{i}|,$$

(22) $$RMSE=\sqrt{\frac{1}{N}\sum_{i=1}^{N}\;(y_{i}-{\hat{y}}_{i}{)}^{2}},$$

where $y_{i}$ is the actual output value of the i−th sample, $\bar{y}$ is the mean of the actual values, ${\hat{y}}_{i}$ is the predicted output value, and N is the total number of samples.

### 4.3. Overview of Experimental Procedures

In this experiment, 1000 samples are used for training, while an additional 30 samples are set aside for testing. The model is configured to undergo 100 training iterations, and the optimization algorithm parameters are provided in Table 8. To balance computational efficiency and algorithm performance, the population size for all algorithms in this study was uniformly set to 30.

**Figure 9:**
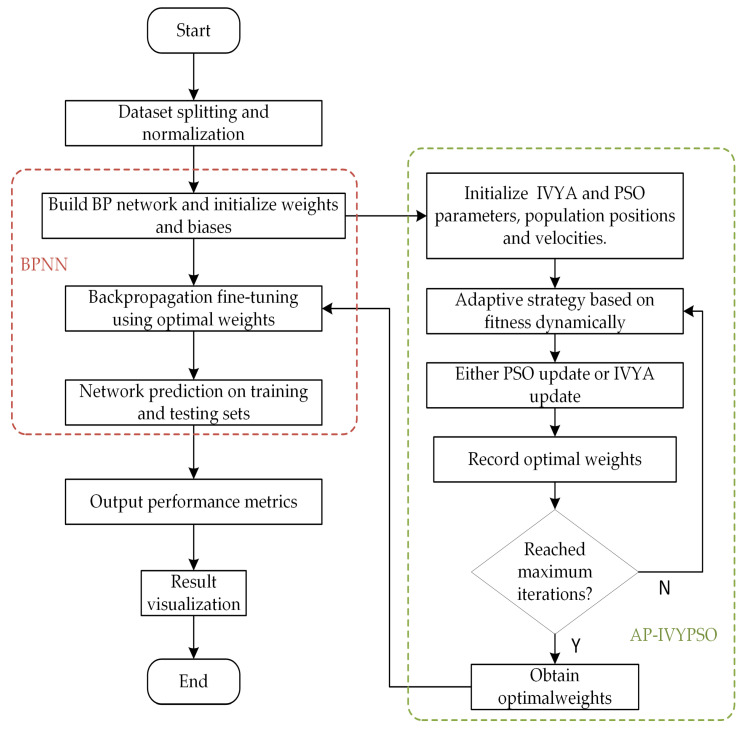
The flowchart of the experimental procedure.

The flowchart of the experimental procedure is shown in Figure 9. The specific procedures for conducting this experiment are outlined as follows.

The specific procedures for conducting this experiment are detailed as follows:

(1) Data Preprocessing and Dataset Division: The original HPC dataset is divided into a training set and a test set. The input features are normalized using the mapminmax function, while the output values are standardized with Z-score normalization. These preprocessing steps help to enhance the model’s stability and speed up convergence during training.
(2) Neural Network Architecture Configuration: A BPNN is constructed, with two hidden layers containing 16 and 8 neurons. The architecture is initialized based on the input features’ dimensionality and the number of output variables.
(3) AP-IVYPSO Initialization: The maximum number of iterations and population size for the AP-IVYPSO algorithm are set. Each “vine” individual represents a potential combination of neural network weights and thresholds, which are the optimization targets. The individual particles dynamically select between the PSO or IVYA update strategies, using an adaptive probability mechanism based on fitness improvements.
(4) Fitness Evaluation: The fitness function is defined as the RMSE between predicted and actual values. This guides the “vine” individuals toward the optimal solution, ensuring that the network’s predictive performance is maximized.
(5) Position Update and Local Search: Each “vine” individual updates its position either by applying a local disturbance strategy or by randomly selecting a leader’s direction. In PSO updates, particle velocity and position are adjusted using the velocity update mechanism. In IVYA updates, the position is modified using vine heuristic growth dynamics. A mutation mechanism with a certain probability is incorporated to increase population diversity and help avoid local optima.
(6) Optimal Weight Selection: After all iterations, the “vine” individual with the best fitness is selected, and its corresponding neural network weights and thresholds are used to update the BPNN.
(7) Model Training and Prediction: The BPNN is trained using the optimal weights obtained from the AP-IVYPSO algorithm. Predictions are made for both the training and test sets, and the model’s performance is evaluated using R^2^, MAE, and RMSE metrics. The results are visualized to assess the model’s accuracy.

### 4.4. Analysis of Compressive Strength Prediction for High-Performance Concrete

To assess the practicality and advantages of the AP-IVYPSO-BP model in predicting the compressive strength of high-performance concrete, we systematically compared it with four benchmark models: the unoptimized standard BPNN, the PSO-optimized PSO-BP model, the GA-optimized GA-BP model, and the IVYA-optimized IVY-BP model. By keeping the input features consistent, different optimization strategies were applied to adjust the BPNN parameters, ensuring a comprehensive and fair comparison of model performance under the same dataset and experimental conditions.

**Figure 10:**
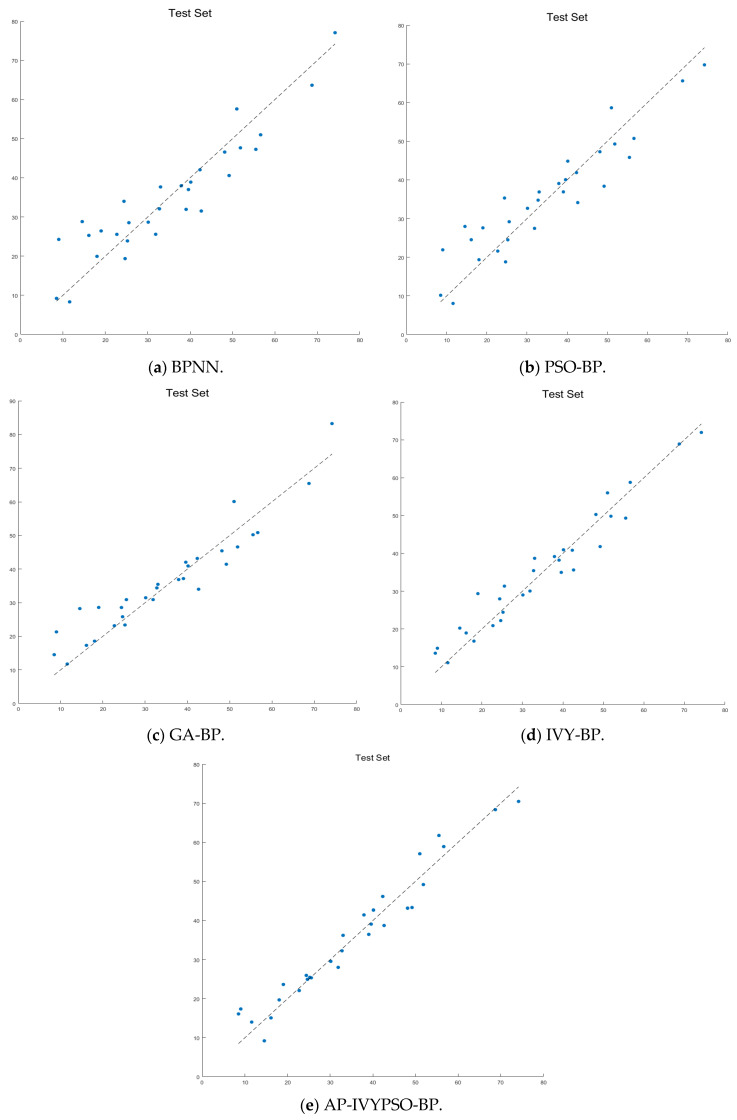
Scatter diagrams comparing predicted and actual test set values across models.

**Figure 11:**
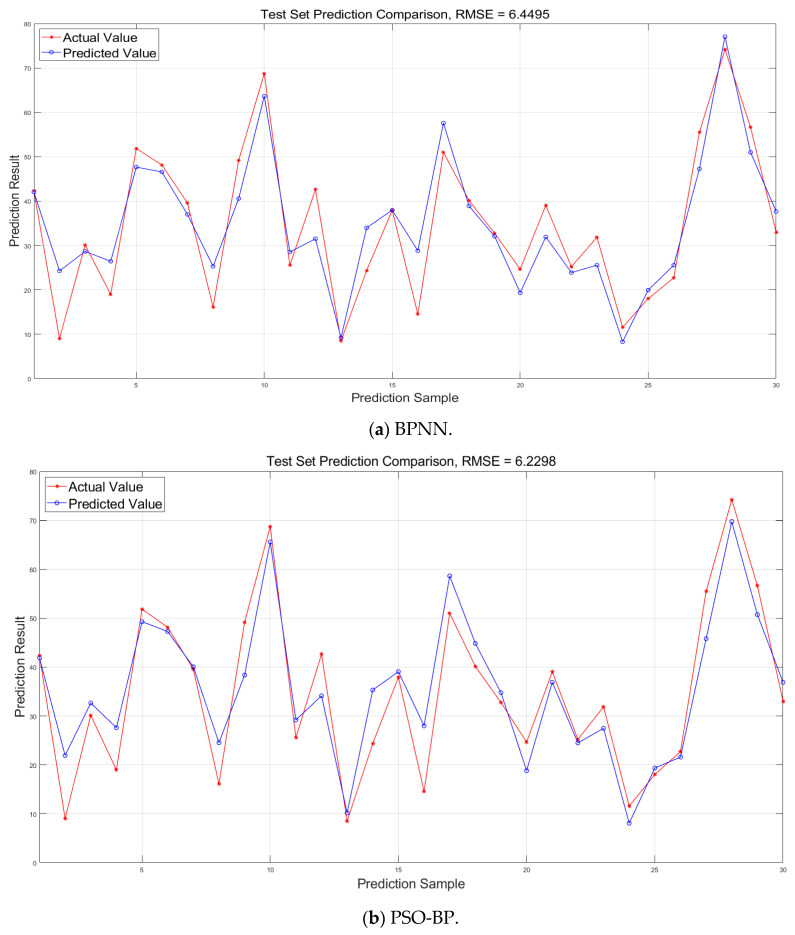

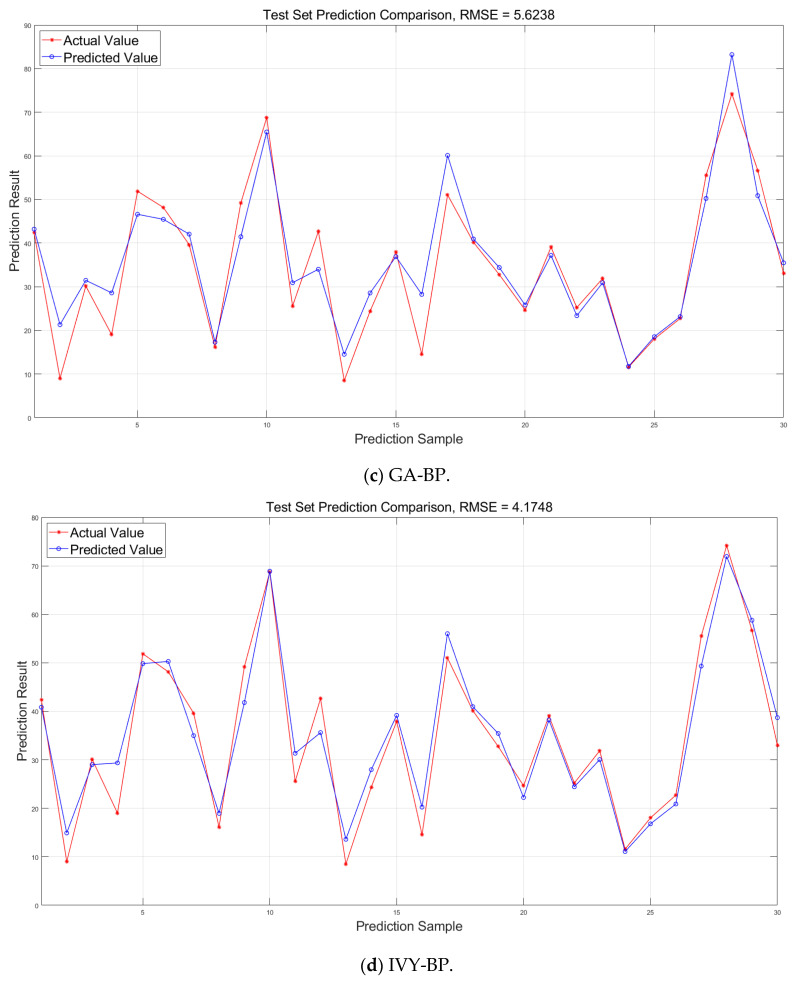

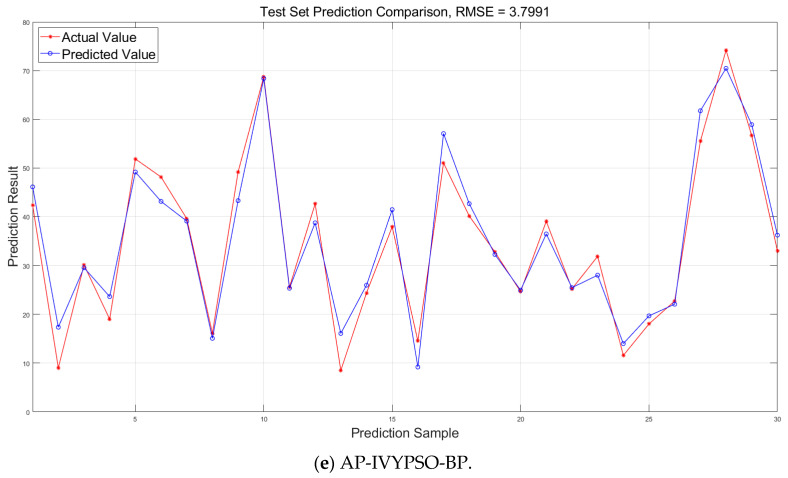
Comparison of predicted and actual values on the test set for different models.

To facilitate a more intuitive comparison of the prediction performance, we present scatter plots (Figure 10) and prediction curves (Figure 11) showing actual values against predicted values. These visualizations demonstrate the performance of the BPNN, PSO-BP, GA-BP, IVY-BP, and AP-IVYPSO-BP models on the test set.

As shown in Figure 10, the scatter plot clearly indicates that the predicted points of the AP-IVYPSO-BP model are the most tightly clustered and almost uniformly distributed along the ideal diagonal (where predicted values equal actual values), suggesting minimal deviation between the actual and predicted values. In contrast, the scatter plots for the BPNN, PSO-BP, GA-BP, and IVY-BP models show greater dispersion, with the BPNN model exhibiting more pronounced prediction errors.

The prediction curve in Figure 11 highlights how well the AP-IVYPSO-BP model fits the entire sample range, with the predicted curve closely matching the actual curve and demonstrating consistent fluctuations and trends. In comparison, other models show clear deviations at certain sample points, particularly at extreme values or inflection points, indicating poor fitting performance. Furthermore, the AP-IVYPSO-BP model excels in tracking regions with large fluctuations in the data, effectively capturing complex nonlinear relationships. This underscores the superior fitting ability and stability of the AP-IVYPSO-BP model when dealing with complex data structures. Table 9 provides the prediction results for each model, from which we can draw the following conclusions. Additionally, the table also provides the runtime (TIME) of each model.

The AP-IVYPSO-BP model outperforms all comparison models, especially in terms of prediction performance on the test set. Specifically, it surpasses the traditional BPNN, PSO-BP, GA-BP, and IVY-BP models in key evaluation metrics such as R^2^, MAE, and RMSE. Notably, it achieves R^2^ = 0.9542, MAE = 3.0404, and RMSE = 3.7991, demonstrating minimal deviation between the predicted and actual values. These results show that the AP-IVYPSO-BP model delivers optimal prediction accuracy for high-performance concrete compressive strength.

Compared to the traditional BPNN model, the PSO and GA algorithms have already enhanced its performance, and the IVYA further improves the predictive capability of BPNN. The AP-IVYPSO-BP model combines the global search ability of PSO with the local search characteristics of IVYA, dynamically adjusting the PSO and IVYA update mechanisms through an adaptive probability strategy based on fitness improvement. This dynamic balance between global and local searches significantly enhances both prediction accuracy and model stability. When compared to the IVY-BP model, which is less optimized, the AP-IVYPSO-BP model improves R^2^ from 0.9485 to 0.9542, reduces MAE by 0.3155, and lowers RMSE by 0.3757. These improvements clearly demonstrate the superior prediction accuracy of the AP-IVYPSO-BP model over other optimization techniques.

In conclusion, these results suggest that integrating bio-inspired optimization algorithms with neural networks is an effective approach for improving regression prediction accuracy in complex nonlinear problems. By leveraging both global and local search capabilities, the AP-IVYPSO-BP model demonstrates remarkable generalization ability and robustness, achieving notable success in predicting the compressive strength of high-performance concrete.

## 5. Conclusions

This paper introduces a novel hybrid prediction model, AP-IVYPSO-BP, that combines the bio-inspired IVYA with PSO to optimize a BPNN for accurately predicting the compressive strength of HPC. The AP-IVYPSO-BP model strengthens the global search capability of PSO and the local search characteristics of IVYA, while dynamically adjusting their update mechanisms through an adaptive probability strategy based on fitness improvement. This dynamic adjustment optimizes the balance between global and local searches, significantly enhancing prediction accuracy, model stability, and robustness.

To validate the proposed model’s effectiveness, experiments were conducted on a publicly available dataset containing 1030 high-performance concrete mix samples. The AP-IVYPSO-BP model was compared with traditional BPNN, PSO-BP, GA-BP, and IVY-BP models. The experimental results demonstrate that the AP-IVYPSO-BP model outperforms the other models across various evaluation metrics, particularly excelling in R^2^, MAE, and RMSE. Specifically, the AP-IVYPSO-BP model achieved an R^2^ of 95.42%, reflecting its excellent fitting ability and prediction accuracy. Moreover, MAE and RMSE showed substantial improvements compared to the baseline models, further highlighting the model’s superior performance in predicting concrete compressive strength.

The AP-IVYPSO-BP model provides an effective tool for accurately predicting concrete strength, contributing to better material utilization, reduced resource waste, and minimized environmental impact, thereby supporting the sustainable development of the construction industry. Future research could explore applying this model to the prediction of other engineering materials’ strength, incorporating additional optimization algorithms and deep learning techniques to further enhance the model’s performance. Furthermore, investigating the model’s practical applications in engineering management will help unlock its full potential for sustainable development.

## Figures and Tables

**Table 1:** Details of the 26 test functions.

s/n	Category	Function Name	Formula	Range	$f_{min}^{*}$
f1	Unimodal	Sphere	$f_{1}(x)=\sum_{i=1}^{dim}\;x_{i}^{2}$	[−100, 100]	0
f2	Unimodal	Schwefel 2.22	$f_{2}\left|x\right|=\prod_{i=1}^{dim}\;\left|x_{i}\right|+\sum_{i=1}^{dim}\;|x_{i}|$	[−10, 10]	0
f3	Unimodal	Schwefel 1.2	$f_{3}(x)=\sum_{i=1}^{dim}\;{\left(\sum_{j=1}^{i}\;x_{j}\right)}^{2}$	[−100, 100]	0
f4	Unimodal	Schwefel 2.21	$f_{4}(x)=\underset{i}{max}\;\{|x_{i}|\},1\leq i\leq dim$	[−100, 100]	0
f5	Unimodal	Step	$f_{5}(x)=\sum_{i=1}^{dim}\;{\left(0.5+x_{i}\right)}^{2}$	[−100, 100]	0
f6	Unimodal	Quartic	$f_{6}(x)=\sum_{i=1}^{dim}\;ix_{i}^{4}+rand$	[−1.28, 1.28]	0
f7	Unimodal	Exponential	$f_{7}(x)=\sum_{i=1}^{dim}\;(e^{x_{i}}-x_{i})$	[−10, 10]	0
f8	Unimodal	Sum power	$f_{8}(x)=\sum_{i=1}^{dim}\;x_{i}^{2}$	[−1, 1]	0
f9	Unimodal	Sum square	$f_{9}(x)=\sum_{i=1}^{dim}\;ix_{i}^{2}$	[−10, 10]	0
f10	Unimodal	Rosenbrock	$f_{10}(x)=\sum_{i=1}^{dim-1}\;\left((x_{i}-1{)}^{2}+100(x_{i+1}-x_{i}^{2}{)}^{2}\right)$	[−5, 10]	0
f11	Unimodal	Zakharov	$f_{11}(x)={\left(\sum_{i=1}^{dim}\;0.5ix_{i}\right)}^{2}+\sum_{i=1}^{dim}\;x_{i}^{2}+{\left(\sum_{i=1}^{dim}\;0.5ix_{i}\right)}^{4}$	[−5, 10]	0
f12	Unimodal	Trid	$f_{12}(x)=\sum_{i=1}^{dim}\;(1-x_{i}{)}^{2}-\sum_{i=2}^{dim}\;x_{i}x_{i-1}$	[−5, 10]	0
f13	Unimodal	Elliptic	$f_{13}(x)=\sum_{i=1}^{dim}\;(10^{6}{)}^{i/(dim-1)}x_{i}^{2}$	[−100, 100]	0
f14	Unimodal	Cigar	$f_{14}(x)=10^{6}\sum_{i=2}^{dim}\;x_{i}^{2}+x_{1}^{2}$	[−100, 100]	0
f15	Fixed	Rastrigin	$f_{15}(x)=\sum_{i=1}^{dim}\;\left(10-10cos(2\pi x_{i})+x_{i}^{2}\right)$	[−5.12, 5.12]	0
f16	Multimodal	NCRastrigin	$f_{16}(x)=\sum_{i=1}^{dim}\;\left(10-10cos(2\pi x_{i})+x_{i}^{2}\right),y_{i}=\left\{\begin{array}{c} x_{i},\;\mathrm{if}\;x_{i}\leq 0.5\\ x_{i}-1,\;\mathrm{otherwise}\; \end{array}\right.$	[−5.12, 5.12]	0
f17	Multimodal	Ackley	$f_{17}(x)=e^{-1}\sum_{i=1}^{dim}\;cos(2\pi x_{i})+20e^{-0.2\sqrt{\frac{1}{dim}\sum_{i=1}^{dim}\;x_{i}^{2}}}+20+e$	[−50, 50]	0
f18	Multimodal	Griewank	$f_{18}(x)=1-\prod_{i=1}^{dim}\;cos\left(\frac{x_{i}}{\sqrt{i}}\right)+\frac{1}{4000}\sum_{i=1}^{dim}\;x_{i}^{2}$	[−600, 600]	0
f19	Fixed	Alpine	$f_{19}(x)=\sum_{i=1}^{dim}\;|{0.1x_{i}+x}_{i}sin(x_{i})|$	[−10, 10]	0
f20	Multimodal	Penalized 1	$f_{20}\left(x\right)=\frac{\pi }{dim}\left\{\sum_{i=1}^{dim-1}\;(y_{i}-1{)}^{2}\left[1+10\sin^{2}\left(\pi y_{i+1}\right)\right]+\;10{sin}^{2}(\pi y_{1})\right.\left.+(y_{dim}-1{)}^{2}\right\}+\sum_{i=1}^{dim}\;u\left(x_{i},10,100,4\right),y_{i}=1+\;\frac{x_{i}+1}{4},u(x_{i},a,k,m)=\left\{\begin{array}{c} k(x_{i}-a{)}^{m},\;x_{i}>a\\ 0,\;\;\;\;\;-a\leq x_{i}\leq a\\ k(-x_{i}-a{)}^{m},\;x_{i}<-a \end{array}\right.$	[−100, 100]	0
f21	Multimodal	Penalized 2	$f_{21}(x)=\sum_{i=1}^{dim-1}\;(x_{i}-1{)}^{2}\left[1+\sin^{2}\left(3\pi x_{i+1}\right)\right]+0.1\left\{{sin}^{2}(3\pi x_{1})\right.+(x_{dim}\;\;\;\;\;\;\;-1{)}^{2}[1+{sin}^{2}(2\pi x_{dim})]\}+\sum_{i=1}^{dim}\;u(x_{i},5,100,4)$	[−100, 100]	0
f22	Fixed	Schwefel	$f_{22}(x)=\sum_{i=1}^{dim}\;x_{i}sin(\sqrt{\left|x_{i}\right|})$	[−100, 100]	0
f23	Multimodal	Lévy	$f_{23}(x)=\sum_{i=1}^{dim}\;(x_{i}-1{)}^{2}[1+{sin}^{2}(3\pi x_{i+1})]+{sin}^{2}(3\pi x_{1})+(x_{dim}\;\;\;\;\;\;\;-1{)}^{2}[1+{sin}^{2}(2\pi x_{dim})]$	[−10, 10]	0
f24	Multimodal	Weierstrass	$f_{24}(x)=\sum_{i=1}^{dim}\;\left(\sum_{k=0}^{k_{max}}\;a^{k}cos(2\pi b^{k}({0.5+x}_{i}))\right)\;\;\;\;\;\;\;-dim\left(\sum_{k=0}^{k_{max}}\;a^{k}cos(\pi b^{k})\right),a=0.5,b=3,k_{max}\;\;\;\;\;\;\;=20$	[−0.5, 0.5]	0
f25	Fixed	Solomon	$f_{25}(x)=1+0.1\sqrt{\sum_{i=1}^{dim}\;x_{i}^{2}}-cos\left(2\pi \sqrt{\sum_{i=1}^{dim}\;x_{i}^{2}}\right)$	[−100, 100]	0
f26	Fixed	Bohachevsky	$f_{26}(x)=\sum_{i=1}^{dim}\;\left(3x_{i}^{2}-0.3cos(3\pi x_{i})\right)$	[−10, 10]	0

**Table 2:** Parameter settings of 11 algorithms.

Algorithm	Parameter	Algorithm	Parameter
ALL	Max iteration = 500; Agents = 30; Runs = 30	BOA	$a=0.1;p=0.6;c_{0}=0.01$
AP-IVYPSO	$C_{1}=C_{2}=2.0;Vmax=0.1;alpha=0.2$ ωmax=0.9;ωmin=0.4	WOA	$\begin{array}{c} a=\mathrm{linear}\;\mathrm{decrease}\;\mathrm{from}\;2\;\mathrm{to}\;0;\\ C=\left[0,2\right];a2=\mathrm{linear}\;\mathrm{decrease}\;\mathrm{from}-1\;\mathrm{to}-2 \end{array}$
PSO	$C_{1}=C_{2}=2;V=\left(-6,6\right);w=(0.2,0.9)$	GOOSE	$S_{W_{min}}=5;S_{W_{max}}=25;\;coe\_min=0.17$
IVY	$beta_{1}=\left[1,1.5\right);GV=[0,1]$	PSOBOA	$p=0.6;{power}_{exponent}=0.1$ senso−ry_modality=0.01
HFPSO	$C_{1}=C_{2}=1.49445;{Vmax}_{coef}=0.1;$ alpha=0.2;beta0=2;gamma=1;m=2;	NSM-BO	$p_{{xgm}_{initial}}=0.03;scab=1.25;scsb=1.3$ rcpp=0.0035;tsgs_factor_max=0.05
HJSPSO	Cmin=0.5;Cmax=2.0;Wmin=0.4; Wmax=0.9;Bta=0.1	FDB-AGSK	${KF}_{pool}=\left[0.1,1.0,0.5,1.0\right];$ ${KR}_{pool}=[0.2,0.1,0.9,0.9]$

**Table 3:** Best fitness and ranking of AP-IVYPSO and other algorithms.

Func.	Metrics	AP-IVYPSO	PSO	IVY	HFPSO	HJSPSO	BOA	WOA	GOOSE	PSOBOA	NSM-BO	FDB-AGSK
f1	Best	0	2.143	0	1.6538 × 10^−5^	6.3891 × 10^−45^	7.5933 × 10^−11^	5.9355 × 10^−88^	0.0109	1.9073 × 10^−61^	0.0004	7.4370 × 10^−94^
	Rank	1	11	1	8	6	7	4	10	5	9	3
f2	Best	0	6.213	0	0.006	1.7830 × 10^−24^	2.8236 × 10^−8^	5.7347 × 10^−55^	110.4415	2.7886 × 10^−29^	0.0011	2.0686 × 10^−63^
	Rank	1	10	1	9	6	7	4	11	5	8	3
f3	Best	0	71.8221	0	2.5028	1.7817 × 10^−16^	5.2076 × 10^−11^	277.7414	1.9139	2.2011 × 10^−59^	0.0589	281.0804
	Rank	1	9	1	8	4	5	10	7	3	6	11
f4	Best	0	2.1369	0	0.0966	1.8662 × 10^−19^	2.6513 × 10^−8^	5.8994	0.1737	2.9378 × 10^−31^	3.3914	2.0974 × 10^−6^
	Rank	1	9	1	7	4	5	11	8	3	10	6
f5	Best	0.0037	0.9778	2.2433	3.8506 × 10^−6^	0.5192	6.5074	0.0809	0.0122	7.0291	0.0009	1.5393
	Rank	3	7	9	1	6	10	5	4	11	2	8
f6	Best	3.4874 × 10^−5^	40.0583	9.3677 × 10^−5^	0.0301	0.0008	0.0017	0.0018	0.2424	0.0005	0.1606	7.2595 × 10^−5^
	Rank	1	11	3	8	5	6	7	10	4	9	2
f7	Best	7.1751 × 10^−66^	0	3.3691 × 10^−33^	7.1751 × 10^−66^	1.0082 × 10^−64^	8.4738 × 10^−13^	7.1751 × 10^−66^	8.2315 × 10^−66^	2.9324 × 10^−13^	7.1751 × 10^−66^	7.1751 × 10^−66^
	Rank	2	1	9	2	8	11	2	7	10	2	2
f8	Best	0	0.0086	0	6.3555 × 10^−14^	8.6067 × 10^−113^	1.1827 × 10^−13^	4.2612 × 10^−116^	2.5647 × 10^−5^	7.7022 × 10^−72^	5.4193 × 10^−24^	1.8839 × 10^−149^
	Rank	1	11	1	8	5	9	4	10	6	7	3
f9	Best	0	20.5078	0	1.7088 × 10^−5^	2.6389 × 10^−48^	7.5159 × 10^−11^	1.4417 × 10^−78^	0.1856	1.1040 × 10^−60^	1.3930 × 10^−5^	1.9045 × 10^−105^
	Rank	1	11	1	9	6	7	4	10	5	8	3
f10	Best	25.6917	1256.7495	26.9908	26.6159	25.3748	28.8438	27.4877	134.8309	28.9915	38.9212	28.7173
	Rank	2	11	4	3	1	7	5	10	8	9	6
f11	Best	0	194.2087	0	2.5069 × 10^−5^	1.6487 × 10^−47^	7.6444 × 10^−11^	4.9707 × 10^−78^	0.1711	3.0846 × 10^−59^	3.6457	1.8917 × 10^−103^
	Rank	1	11	1	8	6	7	4	9	5	10	3
f12	Best	0.6667	90.0749	0.6667	0.6667	0.6667	0.9718	0.6669	1.0268	0.9985	0.6905	0.6686
	Rank	2	11	1	4	3	8	5	10	9	7	6
f13	Best	0	7.3733 × 10^−31^	0	3.7490 × 10^−43^	5.6779 × 10^−177^	4.7441 × 10^−22^	0	1.7220 × 10^−7^	1.0862 × 10^−62^	0	0
	Rank	1	9	1	8	6	10	1	11	7	1	1
f14	Best	0	3.1468 × 10^−19^	0	5.8483 × 10^−28^	8.0021 × 10^−118^	1.0286 × 10^−16^	2.9238 × 10^−121^	4147.5965	2.9735 × 10^−58^	0	1.4151 × 10^−164^
	Rank	1	9	1	8	6	10	5	11	7	1	4
f15	Best	0	1.4621 × 10^−26^	0	2.4789 × 10^−28^	3.9173 × 10^−162^	4.6394 × 10^−22^	2.4224 × 10^−151^	0.0013	7.8454 × 10^−63^	0	3.9790 × 10^−207^
	Rank	1	9	1	8	5	10	6	11	7	1	4
f16	Best	0	116.392	0	56.7134	0	1.4211 × 10^−12^	0	151.8816	1.7053 × 10^−13^	17.9185	0
	Rank	1	10	1	9	1	7	1	11	6	8	1
f17	Best	0	174.1291	0	56	36.9823	126.8702	0	259.005	3.0749 × 10^−12^	9.0496	0
	Rank	1	10	1	8	7	9	1	11	5	6	1
f18	Best	4.4409 × 10^−16^	2.7332	4.4409 × 10^−16^	0.001	3.9968 × 10^−15^	2.8218 × 10^−8^	4.4409 × 10^−16^	17.4731	4.4409 × 10^−16^	1.6501	3.9968 × 10^−15^
	Rank	1	10	1	8	5	7	1	11	1	9	5
f19	Best	0	0.1477	0	1.1175 × 10^−5^	0	8.7103 × 10^−12^	0	0.045	0	0.1929	0
	Rank	1	10	1	8	1	7	1	9	1	11	1
f20	Best	0	4.7182	0	0.0081	2.4683 × 10^−26^	9.4059 × 10^−11^	2.6009 × 10^−55^	7.2385	6.1770 × 10^−22^	0.0003	9.3253 × 10^−66^
	Rank	1	10	1	9	5	7	4	11	6	8	3
f21	Best	0.0003	0.0524	0.0031	1.9709 × 10^−8^	0.008	0.8402	0.005	3.1567	0.9718	1.0872 × 10^−07^	0.0196
	Rank	3	8	4	1	6	9	5	11	10	2	7
f22	Best	2.5661	1.05	2.6862	2.9258 × 10^−7^	0.1515	2.5055	0.1836	0.011	2.4301	0.011	0.1103
	Rank	10	7	11	1	5	9	6	2	8	3	4
f23	Best	0.24	5.9907	1.0393	0.2439	0.2986	12.2412	0.1273	0.6291	15.6423	0.0465	0.0027
	Rank	4	9	8	5	6	10	3	7	11	2	1
f24	Best	0	1.5995	0	2.2204 × 10^−16^	0	4.2802	0	14.0717	0.0005	0	0
	Rank	1	9	1	7	1	10	1	11	8	1	1
f25	Best	0	1.5919	0	0.8955	0.0995	0.8955	0.0995	1.5919	0.0995	4.8752	0.0995
	Rank	1	9	1	7	5	8	3	10	6	11	4
f26	Best	0	20.5778	0	1.0702 × 10^−5^	0	7.0600 × 10^−11^	0	5.7812	0	0.0002	0
	Rank	1	11	1	8	1	7	1	10	1	9	1
Paired rank +/=/−			24/0/2	7/18/1	22/1/3	20/4/2	25/0/1	16/8/2	25/0/1	22/3/1	17/5/4	17/7/2
Avg. rank		1.73	9.35	2.58	8.69	4.62	8.04	4.00	9.35	6.08	6.15	3.62
Overall rank		1	10	2	9	5	8	4	10	6	7	3

**Table 4:** Average fitness, standard deviation, and ranking of AP-IVYPSO and other algorithms.

Func.	Metrics	AP-IVYPSO	PSO	IVY	HFPSO	HJSPSO	BOA	WOA	GOOSE	PSOBOA	NSM-BO	FDB-AGSK
f1	Avg.	0	2.2242	0	2.7201 × 10^−5^	1.6815 × 10^−45^	2.4795 × 10^−73^	2.1691 × 10^−94^	24.3628	7.6307 × 10^−11^	8.1448 × 10^−2^	1.8042 × 10^−60^
	Std.	0	1.1192	0	1.9166 × 10^−5^	5.4431 × 10^−45^	1.0646 × 10^−72^	1.1247 × 10^−93^	72.7317	5.9056 × 10^−12^	0.1366	3.8616 × 10^−60^
	Rank	1	10	1	8	6	4	3	11	7	9	5
f2	Avg.	0	4.3059	0	5.0487 × 10^−3^	3.6512 × 10^−24^	1.6646 × 10^−50^	1.6909 × 10^−61^	311755.5169	2.2675 × 10^−08^	3.3658 × 10^−3^	3.2835 × 10^−29^
	Std.	0	1.0453	0	3.2921 × 10^−3^	1.2900 × 10^−23^	5.2406 × 10^−50^	4.1066 × 10^−61^	1700375.092	7.0180 × 10^−09^	4.1626 × 10^−3^	3.8575 × 10^−29^
	Rank	1	10	1	9	6	4	3	11	7	8	5
f3	Avg.	0	84.3157	0	1.084	9.3012 × 10^−13^	436.7661	398.6451	2.392	5.3594 × 10^−11^	3.685	7.4448 × 10^−60^
	Std.	0	25.1033	0	0.7504	2.6931 × 10^−12^	150.0621	192.5733	0.875	7.2579 × 10^−12^	3.4865	3.1965 × 10^−59^
	Rank	1	9	1	6	4	11	10	7	5	8	3
f4	Avg.	0	1.9148	0	0.1389	2.4045 × 10^−20^	4.7568	4.051	0.2214	2.7187 × 10^−08^	2.5511	7.9000 × 10^−31^
	Std.	0	0.2904	0	5.9634 × 10^−2^	5.9800 × 10^−20^	2.984	3.7145	9.8125 × 10^−2^	2.6012 × 10^−09^	0.6179	8.1858 × 10^−31^
	Rank	1	8	1	6	4	11	10	7	5	9	3
f5	Avg.	3.2141 × 10^−3^	2.1385	0.4415	2.6084 × 10^−6^	0.1802	8.7297 × 10^−2^	0.5135	1.0139 × 10^−2^	5.3699	5.0195 × 10^−3^	6.291
	Std.	1.0477 × 10^−3^	0.8369	0.4196	1.3344 × 10^−6^	0.1579	5.1211 × 10^−2^	0.3403	3.3982 × 10^−3^	0.6426	1.5448 × 10^−2^	0.6757
	Rank	2	9	7	1	6	5	8	4	10	3	11
f6	Avg.	8.3694 × 10^−5^	13.6561	7.6814 × 10^−5^	1.8698 × 10^−2^	1.5350 × 10^−03^	2.7534 × 10^−3^	1.0183 × 10^−3^	0.1333	2.0667 × 10^−03^	0.1027	2.0759 × 10^−4^
	Std.	7.0251 × 10^−5^	11.6369	9.4172 × 10^−5^	8.0492 × 10^−3^	6.1941 × 10^−04^	2.9686 × 10^−3^	2.0364 × 10^−3^	6.3191 × 10^−2^	6.6445 × 10^−04^	3.7157 × 10^−2^	1.2431 × 10^−4^
	Rank	2	11	1	8	5	7	4	10	6	9	3
f7	Avg.	1.5811 × 10^−62^	0	3.6996 × 10^−32^	7.1751 × 10^−66^	2.1876 × 10^−64^	7.1751 × 10^−66^	7.1751 × 10^−66^	1.2778 × 10^−65^	5.7664 × 10^−10^	7.1751 × 10^−66^	3.7060 × 10^−10^
	Std.	4.8221 × 10^−62^	0	1.3245 × 10^−31^	3.2167 × 10^−81^	4.4300 × 10^−64^	3.2167 × 10^−81^	3.2167 × 10^−81^	2.1948 × 10^−65^	2.3165 × 10^−9^	3.2167 × 10^−81^	1.8506 × 10^−9^
	Rank	8	1	9	2	7	3	4	6	11	5	10
f8	Avg.	0	0.1845	0	1.3148 × 10^−13^	1.0941 × 10^−115^	3.9508 × 10^−112^	2.1483 × 10^−148^	1.4983 × 10^−5^	8.2880 × 10^−14^	4.9084 × 10^−20^	7.1843 × 10^−71^
	Std.	0	0.1404	0	2.5209 × 10^−13^	4.1900 × 10^−115^	1.3697 × 10^−111^	1.1765 × 10^−147^	1.0298 × 10^−5^	5.2208 × 10^−14^	2.5907 × 10^−19^	1.9871 × 10^−70^
	Rank	1	11	1	9	4	5	3	10	8	7	6
f9	Avg.	0	24.6964	0	5.8998 × 10^−5^	8.5050 × 10^−46^	3.1248 × 10^−75^	2.8005 × 10^−96^	1.1455	7.2583 × 10^−11^	0.1602	3.3952 × 10^−59^
	Std.	0	10.5308	0	4.1394 × 10^−5^	2.8022 × 10^−45^	1.5279 × 10^−74^	1.4385 × 10^−95^	0.839	7.1158 × 10^−12^	0.617	7.8186 × 10^−59^
	Rank	1	11	1	8	6	4	3	10	7	9	5
f10	Avg.	25.7396	968.8295	26.7298	34.2495	26.657	27.9368	28.7227	52.7595	28.9095	74.3919	28.9655
	Std.	0.295	430.3186	0.7416	20.3032	0.6231	0.4449	5.4142 × 10^−2^	50.1432	2.5496 × 10^−2^	61.2205	2.0377 × 10^−2^
	Rank	1	11	3	8	2	4	5	9	6	10	7
f11	Avg.	0	104.1111	0	2.8299 × 10^−5^	3.8794 × 10^−46^	7.1179 × 10^−75^	4.5743 × 10^−99^	0.1626	6.7352 × 10^−11^	4.3200 × 10^−2^	2.7784 × 10^−59^
	Std.	0	47.2141	0	1.9534 × 10^−5^	1.4895 × 10^−45^	2.8523 × 10^−74^	1.5075 × 10^−98^	6.0467 × 10^−2^	5.9932 × 10^−12^	0.1679	4.4222 × 10^−59^
	Rank	1	11	1	8	6	4	3	10	7	9	5
f12	Avg.	0.6667	188.7117	0.6667	0.7436	0.6667	0.6669	0.7692	2.6967	0.9714	4.0598	0.9945
	Std.	7.9805 × 10^−8^	111.3023	6.4798 × 10^−8^	0.1496	5.7002 × 10^−7^	1.5067 × 10^−4^	0.117	2.3149	7.2435 × 10^−3^	4.2141	4.7855 × 10^−3^
	Rank	1	11	2	5	3	4	6	9	7	10	8
f13	Avg.	0	7.4583 × 10^−26^	0	6.9712 × 10^−35^	2.0885 × 10^−175^	0	0	3.8853 × 10^−4^	7.8267 × 10^−22^	0	1.8237 × 10^−60^
	Std.	0	2.0498 × 10^−25^	0	3.1468 × 10^−34^	0	0	0	5.6794 × 10^−4^	3.9371 × 10^−21^	0	6.9177 × 10^−60^
	Rank	1	9	1	8	6	1	1	11	10	1	7
f14	Avg.	0	6.2909 × 10^−18^	0	9.9752 × 10^−25^	3.3824 × 10^−121^	4.7568 × 10^−108^	3.0927 × 10^−148^	1424.1393	3.8701 × 10^−17^	0	9.4288 × 10^−56^
	Std.	0	1.9893 × 10^−17^	0	3.2933 × 10^−24^	1.2320 × 10^−120^	1.8377 × 10^−107^	1.6939 × 10^−147^	1800.3152	4.8754 × 10^−17^	0	3.6693 × 10^−55^
	Rank	1	9	1	8	5	6	4	11	10	1	7
f15	Avg.	0	1.5805 × 10^−23^	0	2.4574 × 10^−23^	1.6178 × 10^−160^	9.1804 × 10^−129^	4.5201 × 10^−182^	1.3062 × 10^−2^	8.1037 × 10^−19^	0	4.5225 × 10^−62^
	Std.	0	5.2171 × 10^−23^	0	1.2322 × 10^−22^	5.4692 × 10^−160^	4.0309 × 10^−128^	0	3.7231 × 10^−2^	3.2740 × 10^−18^	0	1.3610 × 10^−61^
	Rank	1	8	1	9	5	6	4	11	10	1	7
f16	Avg.	0	160.4583	0	55.2544	0.758	0	1.8948 × 10^−15^	160.4137	19.6471	12.1124	1.5460 × 10^−10^
	Std.	0	29.6627	0	23.9875	4.1516	0	1.0378 × 10^−14^	41.0324	59.2622	5.3807	7.9237 × 10^−10^
	Rank	1	11	1	9	6	1	4	10	8	7	5
f17	Avg.	0	173.302	0	60.7	20.4006	0	0	180.996	99.7438	8.5838	1.0908 × 10^−7^
	Std.	0	34.9327	0	18.9303	10.6438	0	0	44.8631	83.3625	2.7231	4.2793 × 10^−7^
	Rank	1	10	1	8	7	1	1	11	9	6	5
f18	Avg.	4.4409 × 10^−16^	2.6323	4.4409 × 10^−16^	0.1419	3.9968 × 10^−15^	3.5231 × 10^−15^	2.8126 × 10^−15^	8.8537	2.7313 × 10^−8^	0.8815	4.4409 × 10^−16^
	Std.	0	0.3714	0	0.4423	0	2.4210 × 10^−15^	2.1546 × 10^−15^	7.4732	2.1311 × 10^−9^	0.6703	0
	Rank	1	10	1	8	6	5	4	11	7	9	1
f19	Avg.	0	0.1393	0	1.4138 × 10^−2^	0	2.1449 × 10^−2^	0	254.9518	1.0409 × 10^−11^	0.2475	0
	Std.	0	5.2798 × 10^−02^	0	1.8405 × 10^−2^	0	5.6326 × 10^−2^	0	215.8963	8.5551 × 10^−12^	0.3513	0
	Rank	1	9	1	7	1	8	1	11	6	10	1
f20	Avg.	0	5.0555	0	1.3143 × 10^−2^	3.1090 × 10^−25^	7.7033 × 10^−39^	2.4542 × 10^−61^	6.5153	9.4890 × 10^−9^	8.4556 × 10^−4^	3.4163 × 10^−19^
	Std.	0	2.4515	0	1.1284 × 10^−2^	3.7341 × 10^−25^	4.2193 × 10^−38^	1.1818 × 10^−60^	2.5439	1.7541 × 10^−8^	7.7548 × 10^−4^	1.8447 × 10^−18^
	Rank	1	10	1	9	5	4	3	11	7	8	6
f21	Avg.	2.6129 × 10^−4^	6.4649 × 10^−2^	1.8404 × 10^−2^	0.0276	3.4536 × 10^−3^	2.4664 × 10^−2^	1.6021 × 10^−2^	3.5507	0.5319	1.7249 × 10^−2^	0.8967
	Std.	9.6524 × 10^−5^	9.1396 × 10^−2^	1.5129 × 10^−2^	6.0465 × 10^−2^	4.3030 × 10^−3^	7.8161 × 10^−2^	1.3305 × 10^−2^	1.0116	0.1661	3.9212 × 10^−2^	0.1935
	Rank	1	8	5	7	2	6	3	11	9	4	10
f22	Avg.	2.4622	0.5032	2.8295	4.0291 × 10^−3^	0.3516	0.1426	0.1419	1.0274 × 10^−2^	2.841	7.7291 × 10^−3^	2.8331
	Std.	0.9841	0.2057	0.4535	5.3853 × 10^−3^	0.258	7.9898 × 10^−2^	0.1008	7.5354 × 10^−3^	0.2182	1.1225 × 10^−2^	0.2424
	Rank	8	7	9	1	6	5	4	3	11	2	10
f23	Avg.	0.6783	6.2706	1.2216	0.3475	0.3146	0.5131	0.2782	0.7417	11.7537	4.5073 × 10^−2^	16.2517
	Std.	0.6903	3.2296	1.1141	1.05	0.3326	0.9707	0.3892	0.5245	2.3392	6.6888 × 10^−2^	2.4806
	Rank	6	9	8	4	3	5	2	7	10	1	11
f24	Avg.	0	4.9842	2.4961 × 10^−6^	0.1789	0	0	0	10.3472	0.874	3.1999 × 10^−3^	5.4261 × 10^−5^
	Std.	0	4.2589	1.3672 × 10^−5^	0.6958	0	0	0	5.5363	2.2389	1.1460 × 10^−2^	1.1400 × 10^−4^
	Rank	1	10	5	8	1	1	1	11	9	7	6
f25	Avg.	0	1.879	0	0.9452	9.9496 × 10^−2^	0.1824	9.2863 × 10^−2^	1.6019	0.8031	5.9099	0.0995
	Std.	0	0.5026	0	0.3394	1.9179 × 10^−8^	0.1457	0.1137	0.4884	0.1906	2.0133	1.9888 × 10^−6^
	Rank	1	10	1	8	4	6	3	9	7	11	5
f26	Avg.	0	22.0237	0	0.3632	0	0	0	5.3291	7.5784 × 10^−11^	0.4015	0
	Std.	0	5.5998	0	0.6823	0	0	0	2.1236	6.8529 × 10^−12^	0.912	0
	Rank	1	11	1	8	1	1	1	10	7	9	1
Paired rank +/=/−			24/0/2	8/19/1	22/0/4	20/3/3	18/5/3	18/5/3	24/0/2	26/0/0	20/3/3	23/0/3
Avg. rank		1.81	9.38	2.54	6.92	4.50	4.69	3.77	9.31	7.92	6.65	5.88
Overall rank		1	11	2	8	4	5	3	10	9	7	6

**Table 5:** Friedman ranking of each algorithm.

Algorithm	Scores	Rank
AP-IVYPSO	1.8587	1
PSO	9.2135	10
IVY	2.6871	2
HFPSO	6.7202	8
HJSPSO	4.4745	4
BOA	4.6521	5
WOA	5.8786	6
GOOSE	9.236	11
PSOBOA	7.931	9
NSM-BO	6.6456	7
FDB-AGSK	3.7476	3

**Table 6:** Wilcoxon signed-rank test results for AP-IVYPSO and the other eight algorithms with α = 0.05.

Algorithm	*p*-Value	Significant
AP-IVYPSO-PSO	0.00005	Yes
AP-IVYPSO-IVY	0.01088	Yes
AP-IVYPSO-HFPSO	0.00187	Yes
AP-IVYPSO-HJSPSO	0.0105	Yes
AP-IVYPSO-BOA	0.00117	Yes
AP-IVYPSO-WOA	0.01614	Yes
AP-IVYPSO-GOOSE	0.00003	Yes
AP-IVYPSO-PSOBOA	0.00679	Yes
AP-IVYPSO-NSM-BO	0.00051	Yes
AP-IVYPSO-FDB-AGSK	0.02367	Yes

**Table 7:** Statistical information for HPC datasets.

Type	Variable	Minimum	Maximum Value	Average Value	Standard Deviation	Unit
input variables	cement	102	540	281.1	104.54	Kg/m^3^
	blast furnace slag	0	359.4	73.97	86.29	Kg/m^3^
	fly ash	0	200.1	54.24	64.01	Kg/m^3^
	water	121.8	247	181.55	21.35	Kg/m^3^
	superplasticizer	0	32.2	6.21	5.97	Kg/m^3^
	coarse aggregate	801	1145	972.92	77.79	Kg/m^3^
	fine aggregate	594	992.6	773.58	80.21	Kg/m^3^
	age	1	365	45.62	63.19	days
output variable	compressive strength	2.33	82.6	35.82	16.71	MPa

**Table 8:** Parameter settings of BP, PSO-BP, GA-BP, and IVY-BP.

Model	Parameter Setting	Model	Parameter Setting
BP	Epochs=1000 Error Goal=0.000001 Learning Rate=0.01	PSO-BP	$C_{1}=C_{2}=4.494$ V=(−1,1),ω=0.2
GA-BP	Selection pressure=0.09 Crossover Rate=2 Mutation Rate=[2,50,3]	IVY-BP	$N=50,\alpha =0.9\times \left(1-\frac{t}{MaxIter}\right)$ GV=[0,1]
AP-IVYPSO-BP	$C_{1}=C_{2}=2.0,Vmax=0.1,alpha=0.2,\omega max=0.9,\omega min=0.4$		

**Table 9:** Prediction results of different models.

Index	Model				
	BPNN	PSO-BP	GA-BP	IVY-BP	AP-IVYPSO-BP
$R^{2}$	0.8533	0.8631	0.8885	0.9385	0.9542
MAE	5.08	4.9142	4.2209	3.3559	3.0404
RMSE	6.4495	6.2298	5.6238	4.1748	3.7991
TIME	0.4466	0.8946	0.9136	0.7836	0.9667

## Data Availability

All data used and/or analyzed during this research are openly available and can be accessed freely. If needed, they can be requested from the corresponding author.
